# Enamel peptides reveal the sex of the Late Antique ‘Lovers of Modena’

**DOI:** 10.1038/s41598-019-49562-7

**Published:** 2019-09-11

**Authors:** Federico Lugli, Giulia Di Rocco, Antonino Vazzana, Filippo Genovese, Diego Pinetti, Elisabetta Cilli, Maria Cristina Carile, Sara Silvestrini, Gaia Gabanini, Simona Arrighi, Laura Buti, Eugenio Bortolini, Anna Cipriani, Carla Figus, Giulia Marciani, Gregorio Oxilia, Matteo Romandini, Rita Sorrentino, Marco Sola, Stefano Benazzi

**Affiliations:** 1 Department of Cultural Heritage, University of Bologna, Via degli Ariani 1, 48121 Ravenna, Italy; 2 Department of Chemical and Geological Sciences, University of Modena and Reggio Emilia, Via Campi 103, 41125 Modena, Italy; 3 Department of Life Sciences, University of Modena and Reggio Emilia, Via Campi 103, 41125 Modena, Italy; 4 Centro Interdipartimentale Grandi Strumenti, University of Modena and Reggio Emilia, Via Campi 213/A, 41125 Modena, Italy; 5 Lamont-Doherty Earth Observatory, Columbia University, Palisades, New York, USA; 6 Department of Biological, Geological and Environmental Sciences, University of Bologna, 40126 Bologna, Italy; 7 Department of Human Evolution, Max Planck Institute for Evolutionary Anthropology, Leipzig, Germany

**Keywords:** Proteomics, Anthropology

## Abstract

Recent work has disclosed the critical role played by enamel peptides in sex classification of old skeletal remains. In particular, protein AMELY (amelogenin isoform Y) is present in the enamel dental tissue of male individuals only, while AMELX (isoform X) can be found in both sexes. AMELY can be easily detected by LC-MS/MS in the ion extracted chromatograms of the SM_(ox)_IRPPY peptide (monoisotopic [M + 2 H]^+2^ mass = 440.2233 *m/z*). In this paper, we exploited the dimorphic features of the amelogenin protein to determine the sex of the so-called ‘Lovers of Modena’, two Late Antique individuals whose skeletons were intentionally buried hand-in-hand. Upon discovery, mass media had immediately assumed they were a male-female couple, even if bad preservation of the bones did not allow an effective sex classification. We were able to extract proteins from the dental enamel of both individuals (~1600 years old) and to confidently classify them as males. Results were compared to 14 modern and archaeological control samples, confirming the reliability of the ion chromatogram method for sex determination. Although we currently have no information on the actual relationship between the ‘Lovers of Modena’ (affective? Kin-based?), the discovery of two adult males intentionally buried hand-in-hand may have profound implications for our understanding of funerary practices in Late Antique Italy.

## Introduction

Sex, together with age-at-death and height, is one of the critical pieces of information needed to define the biological profile of skeletal remains. Although some bones are sexually dimorphic (*e.g*., cranium and *os coxae*)^1,2^, the bad state of preservation of some archaeological remains might alter or totally compromise the readability of dimorphic traits in an individual, even if the appropriate osteological techniques are applied. Similarly, taxonomy, context and/or age may also influence the correct determination of sex^3–5^. For example, it is usually straightforward to determine the sex of a buried adult human through the macroscopic examination of dimorphic districts, while it can be difficult to ascertain the sex of faunal remains or of pre-pubertal individuals^4^. In these contexts, DNA may be a valid alternative for sex determination even if the analytical costs and the survival of the DNA itself may strongly limit the use of genetic markers^6^.

Recently, a paper by Stewart *et al*.^7^ has revolutionized the way to achieve sex determination of skeletons from archaeological and forensic contexts, thanks to the enamel proteins. One of these proteins is amelogenin, whose gene is translated into two isoforms linked to sexual chromosomes^8^, namely AMELX (present in both sexes) and AMELY (restricted to the male sex only), which constitute 90% of the entire enamel proteome^9^. The homonymous genes are expressed as amelogenins, the proteins within tooth enamel^10^. These proteins are released by ameloblasts (*i.e*., enamel growth cells) during the secretory stage of enamel development^9^, helping the crystal structure organization.

By liquid-chromatography mass spectrometry (LC-MS/MS), a relatively rapid and cost-effective technique, it is possible to check the occurrence of AMELX and/or AMELY isoforms within enamel samples in order to rapidly estimate the sex of an individual. Specifically, the peptide SM_(ox)_IRPPY (monoisotopic [M + 2 H]^+2^ mass 440.2233 *m/z*), is present only in the AMELY isoform, unambiguously characterizing male sex. Conversely, the absence of the AMELY peptides can be due to female sex or to a partial lack of sequence coverage^11^. For this reason, the probability of a female true positive sex estimation is rarely near 100%.

**Figure 1:**
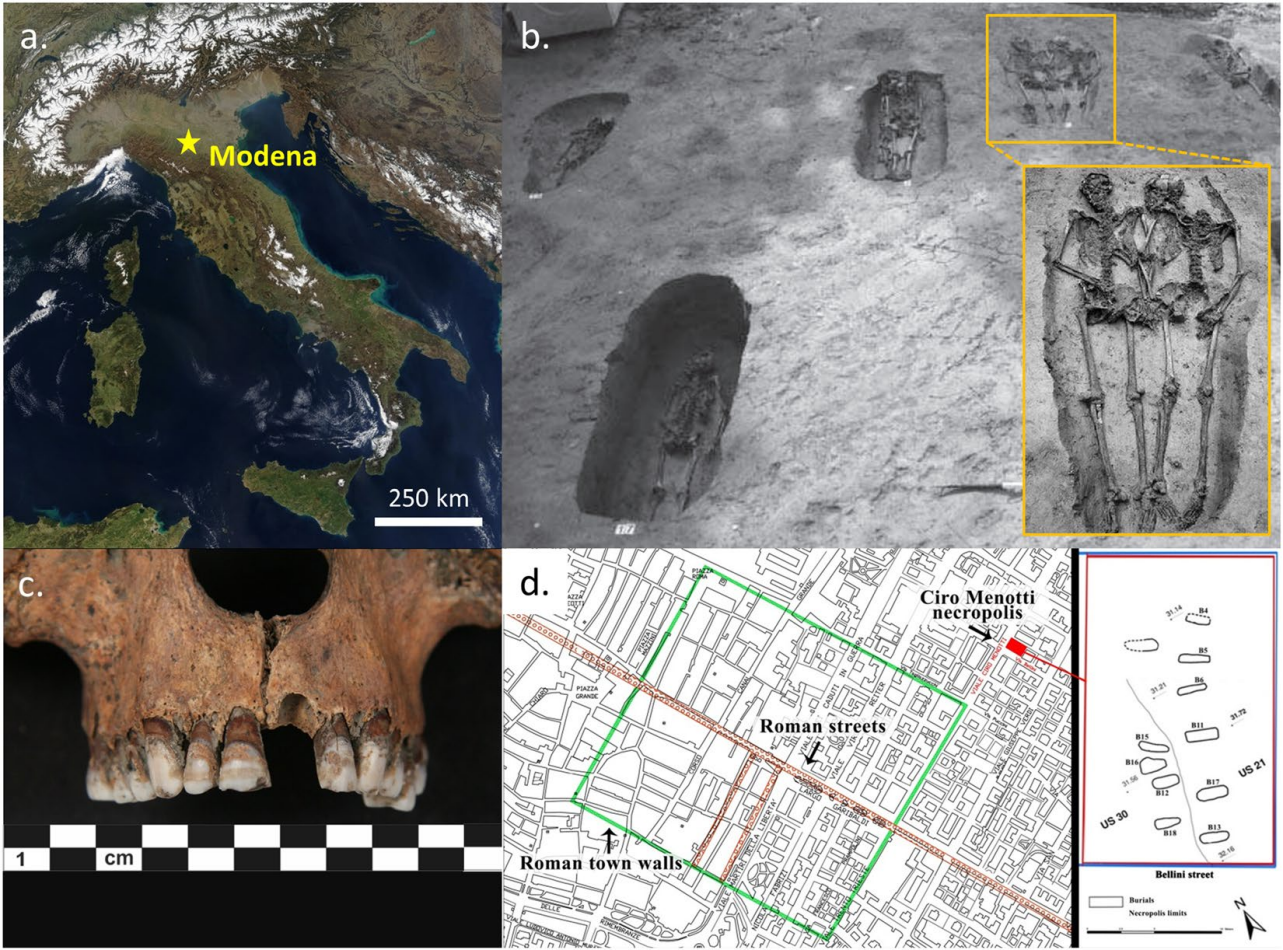
(**a**) Map of Italy with the location of the city of Modena (from NASA Visible Earth project ‒ credits to Jacques Descloitres, MODIS Rapid Response Team, NASA/GSFC); (**b**) Photograph of the Ciro Menotti necropolis (4th–6th century; Modena) during the archaeological excavation; the ‘Lovers of Modena’ are depicted within the inset; (**c**) Teeth and maxilla of individual 7_CM13; (**d**) Roman town of *Mutina* (Latin name of Modena) with the necropolis plan (modified from^12^).

In this work, we examined the enamel proteome of two skeletons exhumed from a peculiar archaeological context, exploiting the sex-specificity of AMELY. In particular, we determined the sex of the so-called ‘Lovers of Modena’, two adult individuals intentionally buried hand-in-hand (Fig. 1; see Figs S17 and^12^). They were found in an Italian Late Antiquity cemetery (4^th^‒6^th^ century, Ciro Menotti, Modena)^12^, together with other eleven individuals. Some of these skeletons showed signs of trauma, likely related to their violent death during war conflicts^12^. Immediately after the discovery of the ‘Lovers’ tomb, news about this peculiar finding spread all over the world and mass media rumored that the skeletons belonged to a man and a woman who had fallen in love^13^. However, due to the bad preservation of the skeletal dimorphic districts, sex determination based on the canonical osteological methods was not possible^14^. Moreover, preliminary genetic analyses were not consistent, due to diagenetic alterations of bone tissue, visible as a black-reddish coloration (see Fig. 1c)^15^, and low collagen content. Hence, we investigated the sex classification of the ‘Lovers’ using enamel peptides, as this tissue is more resistant to diagenetic modifications in both its organic^16^ and inorganic^17,18^ components (see also^19^). Our results were compared to control samples from the same site (n = 2) and from other Italian funerary contexts (n = 10). Two modern deciduous teeth were also used to further check the reliability of the method. To strengthen the method for sex estimation by ion chromatograms^7^, we propose the use of three-peptide peaks, with the aim of confidently identifying AMELY.

## Mass Spectrometry Results

**Figure 2:**
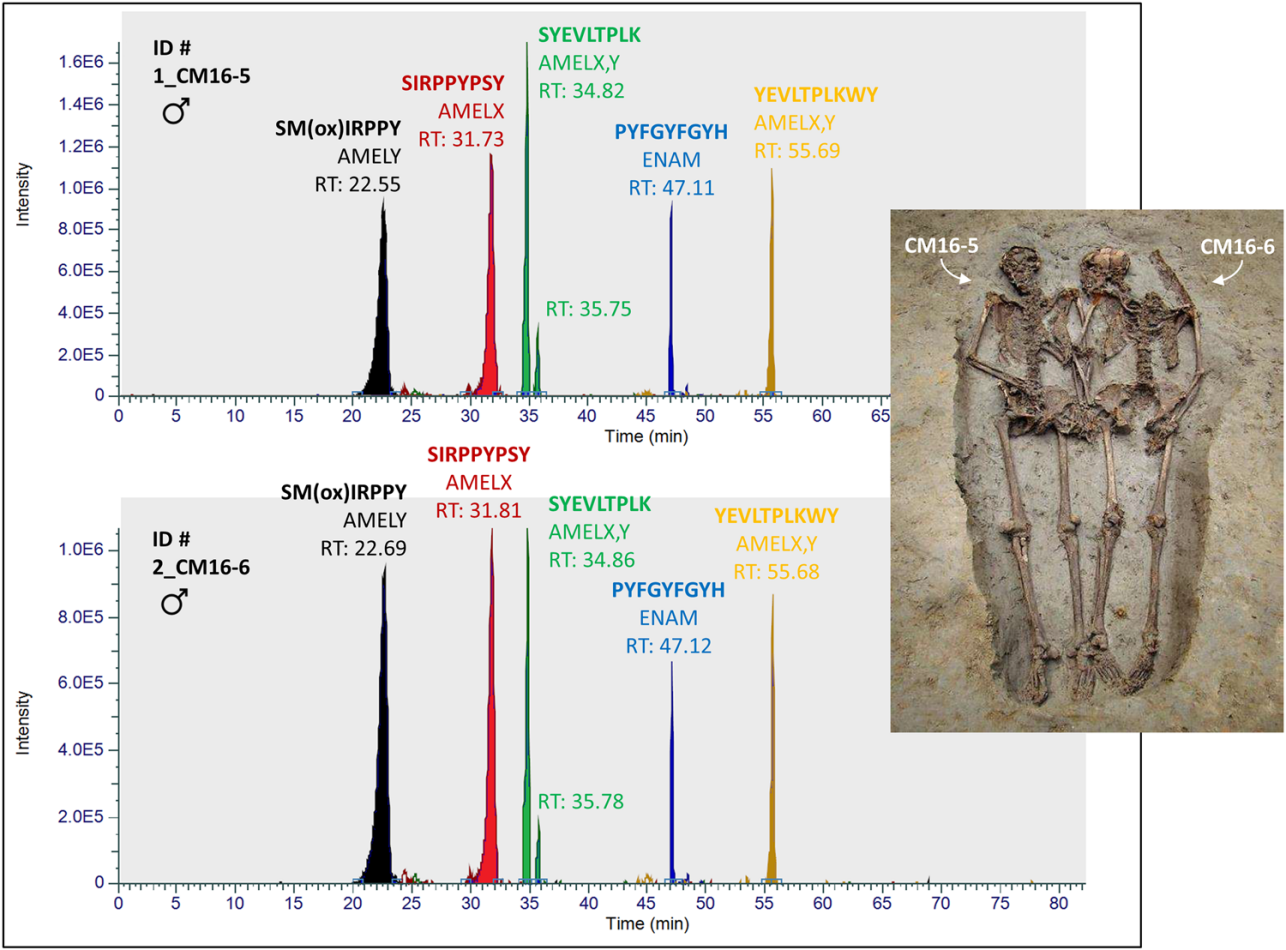
Ion chromatograms representing selected peptides of the ‘Lovers’ enamel proteome^20^. Chromatograms search was performed using Xcalibur software (Thermo Scientific) with a mass tolerance of 5 ppm. Peptide sequences, protein names and retention times are reported in the graphs. The presence of peptide SM_(ox)_IRPPY (AMELY; [M + 2 H]^+2^ 440.2233 *m/z*) in both the specimens suggests that the two individuals were males.

**Figure 3:**
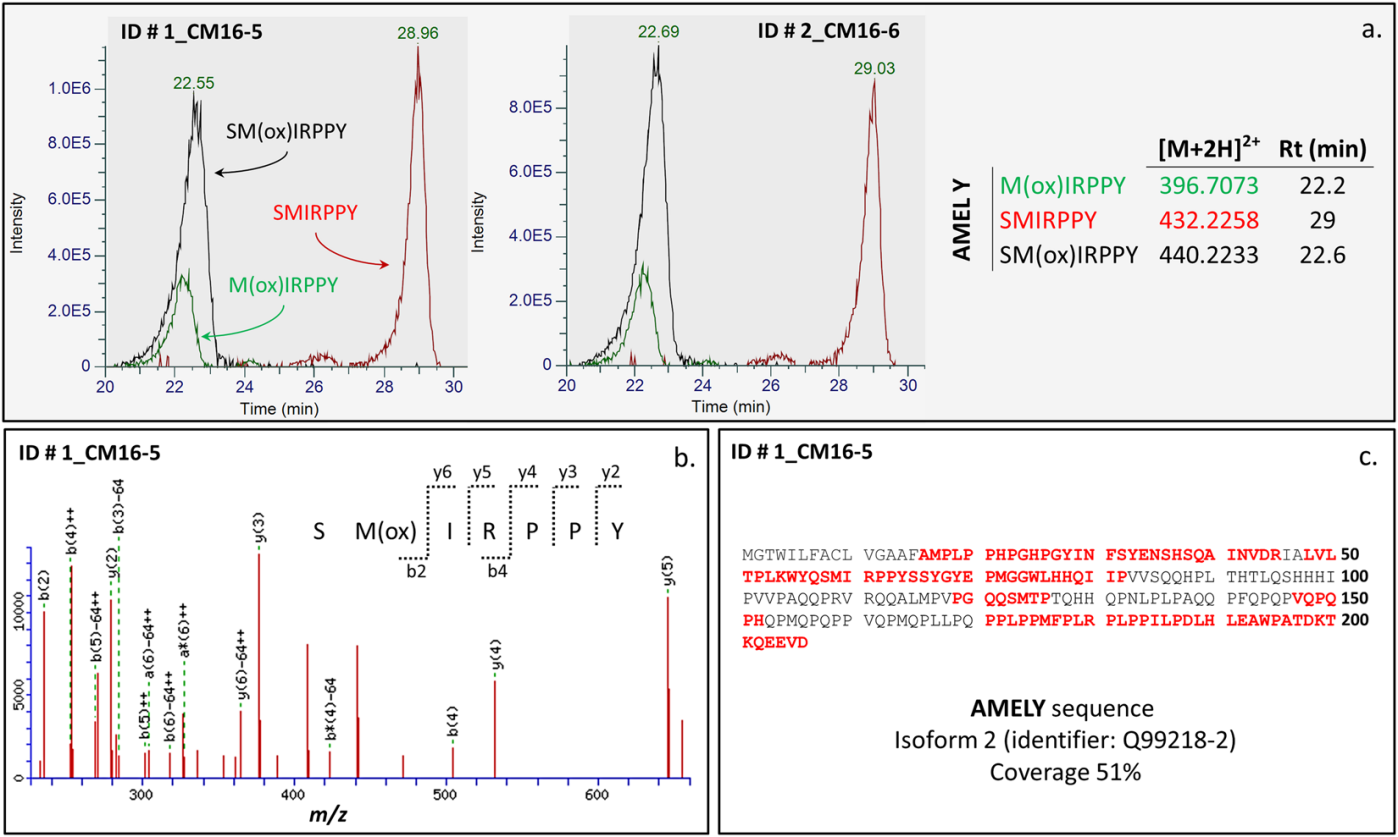
(**a**) Ion chromatogram representing peptides SM(ox)IRPPY, M(ox)IRPPY and SMIRPPY of individual 1_CM16-5 and 2_CM16-6; the occurrence of these three specific peaks confirms the presence of AMELY; (**c**) fragmentation spectrum of peptide SM(ox)IRPPY (monoisotopic mass [M + 2 H]^+2^ 440.2233 *m/z)*; y-axis indicates ion intensities; (**c**) sequence coverage (51%) of AMELY isoform 2 for individual 1_CM16-5.

In total, sixteen human teeth (Table 1) were analyzed during this study. Mascot database search and identification of the extracted peptides revealed that the most detectable species originated from the major tooth enamel proteins amelogenin, enamelin, and ameloblastin (Table S1). Three specimens (4_CM11; 5_FC176 and 17_MdT-2) showed the presence of CO1A2 (human collagen alpha-2(I) chain), likely related to residual dentine tissue. The high resolution (ion tolerance 5 ppm) extracted ion chromatograms for all the teeth revealed the presence of peptides derived from enamel proteins (see Figs S1–S16), as described in^20^: SIRPPYPSY (AMELX; [M + 2 H]^+2^ 540.2796* m/z*); SYEVLTPLK (AMELX,Y; [M + 2 H]^+2^ 525.2975* m/z*); PYFGYFGYH (ENAM; [M + 2 H]^+2^ 575.7533* m/z*); YEVLTPLKWY (AMELX,Y; [M + 2 H]^+2^ 656.3528* m/z*). A subset of the specimens also showed the presence of peptide SM(ox)IRPPY (AMELY; [M + 2 H]^+2^ 440.2233* m/z*), related to male sex^7^. We identified other peptides containing methionine and present only in AMELY, namely M(ox)IRPPY ([M + 2 H]^+2^ 396.7073 *m/z*), SMIRPPY ([M + 2 H]^+2^ 432.2258 *m/z*), SM(ox)RPPYS ([M + 2 H]^+2^ 483.7393 *m/z*) and VLTPLKWYESM(ox)IRPPY ([M + 3 H]^+3^ 670.3570 *m/z*) (see Fig. S18 and Table S2). For all the individuals but one (3_CM12-2), sex estimation based on the osteology agrees with the peptide fingerprint. Individual 3_CM12-2 was initially classified as a young woman through the *ox coxae* and the skull, but the presence of AMELY in its enamel likely suggests that the sex was misclassified. The two modern deciduous teeth were correctly classified as belonging to a male (16_MdT-1) and a female (17_MdT-2) through their enamel peptides (Table 1). Moreover, the presence of AMELY isoform in the enamel of the two ‘Lovers of Modena’ indicates that they were both males (Fig. 2). The occurrence of peptides SMIRPPY, M(ox)IRPPY and SM(ox)IRPPY in ion chromatograms of individuals 1_CM16-5 and 2_CM16-6 confirms the presence of AMELY (Fig. 3a). The fragmentation spectrum of peptide SM(ox)IRPPY from individual 1_CM16-5 is reported as an example (Fig. 3b). For this individual, protein identification through database search of MS^2^ spectra yielded a strong match for AMELY, with a significant protein sequence coverage of 51% (Fig. 3c), unambiguously supporting our hypothesis. Other MS^2^ spectra are reported within the Supplementary Information (see Figs S22–S32).

**Table 1:** Details of the specimens investigated in this study.

LAB ID #	Site location	Period	Burial no.	Sampled tooth	Sex and age*	Sex by enamel peptides	Reference
1_CM16-5	Modena, Ciro Menotti, Emilia Romagna, Italy (‘Lover’)	4th–6th century AD	16.5	Lm^1^	Not determined adult	Male	^12^
2_CM16-6	Modena, Ciro Menotti, Emilia Romagna, Italy (‘Lover’)	4th–6th century AD	16.6	Rm_1_	Not determined adult	Male	^12^
3_CM12-2	Modena, Ciro Menotti, Emilia Romagna, Italy	4th–6th century AD	12.2	Rm^1^	*Female* ‒ *young adult*	*Male*	^12^
4_CM11	Modena, Ciro Menotti, Emilia Romagna, Italy	4th–6th century AD	11.1	Lm^1^	Male ‒ adult	Male	^12^
5_FC176	Forlì Campus, Forlì, Emilia Romagna, Italy	18th century AD	176.1	Rp^3^	Female ‒ adult	Female	—
7_CM13	Modena, Ciro Menotti, Emilia Romagna, Italy	4th–6th century AD	13.1	Lm^2^	Male ‒ adult	Male	^12^
8_PGM11	Modena, Piazza Grande, sector A, Emilia Romagna, Italy	6th–9th century AD	11.1	Lm^3^	Male ‒ adult	Male	^27^
9_PGM17	Modena, Piazza Grande, sector A, Emilia Romagna, Italy	6th–9th century AD	17.1	Lm^2^	Female ‒ adult	Female	^27^
10_PGM13	Modena, Piazza Grande, sector D, Emilia Romagna, Italy	9th–12th century AD	13.1	Lp_4_	Female ‒ adult	Female	^27^
11_PGM12	Modena, Piazza Grande, sector D, Emilia Romagna, Italy	9th–12th century AD	12.1	Rm^2^	Male ‒ young adult	Male	^27^
12_S554	Suasa, Oriental necropolis, Marche, Italy	3th–4th century AD	554.1	Rm_3_	Male ‒ old adult	Male	^28^
13_S562	Suasa, Oriental necropolis, Marche, Italy	1th–2th century AD	562.1	Lp_4_	Female ‒ adult	Female	^28^
14_S564	Suasa, Oriental necropolis, Marche, Italy	2th–3th century AD	564.1	Lm^3^	Female ‒ old adult	Female	^28^
15_S566	Suasa, Oriental necropolis, Marche, Italy	2th–3th century AD	566.1	Rm_3_	Male ‒ old adult	Male	^28^
16_MdT-1	Modena, Italy	Modern (20th century AD)	—	dm^1^	Male ‒ ca. 10 years	Male	—
17_MdT-2	Modena, Italy	Modern (20th century AD)	—	di^1^	Female ‒ ca. 7 years	Female	—

*Sex was determined by pelvis and skull^2,29^; age was determined combining the methods described in^29–32^.

**Figure 4:**
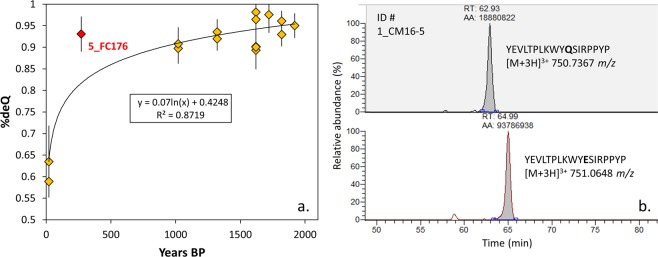
(**a**) Deamidation rate of Gln calculated as reported in the Method section; error bars are 1σ. Sample 5_FC176 seems to be an outlier of the regression; (**b**) example of ion peak integrated area (AA) for peptide YEVLTPLKWYQSIRPPYP, employed for the calculation of %deQ.

To speculate on the age and the post-depositional preservation of enamel samples, we checked the rate of glutamine deamidation, a post-translational modification which involves the conversion of a glutamine (Gln) amino acid residue into glutamic acid, with an expected mass shift of +0.984 Da. Extreme environmental conditions (*e.g*., high temperature, pH and humidity) may accelerate protein deamidation, even though the strong mineral binding allows better preservation and survival of the protein itself ^11,19^. Previous work on collagen, clearly indicates that Gln deamidation strongly correlates with the thermal age of the specimens (which accounts for the burial environmental conditions) rather than the absolute chronological age^21^. The Gln deamidation rate of our samples was checked on those peptides containing the sequence YQSIRPPYP, namely Q57 (see^11^; Fig. 4). We chose to focus on Q57 to possibly compare our data with the previous study of Parker *et al*.^11^; here they also observed that the rate of deamidation at Q57 is slower than in other residue positions^11^. As expected, modern teeth showed a lower degree of %deQ (~0.6) while older samples (up to ~2000 years old) had a higher degree of deamidation (>0.8). Some specimens presented a %deQ close to 1 (completely deamidated), as in the case of 3_CM12-2 (4^th^‒6^th^ cent.) and 12_S554 (3^rd^‒4^th^ cent.). When the %deQ is plotted against the age of the sample (years before present), the dataset seems to fit a logarithmic model (R^2^ = 0.87, p < 0.01; Fig. 4). The only sample presenting a higher degree of deamidation in relation to its age is 5_FC176 (18^th^ cent.), with a %deQ of 0.93 ± 0.03 (residual = 0.11; z-score = 3.0; Fig. S19). Parker *et al*.^11^ state that the rate of deamidation cannot be used to properly check the age of the sample, mostly due to the high rate of variations they observed within samples of the same age. Our samples showed a lower degree of deamidation variability compared to Parker *et al*.^11^, even if specimen 5_FC176 seems to be an outlier. High temperature and low pH may affect deamidation rate, in post-depositional environment, but also during sample pre-treatment^11^. Most studies (see^7,11^ and^16^) use HCl (3–5%) to extract enamel proteins, possibly causing Gln deamidation. The main difference between our study and Parker *et al*.^11^ is the fact that we extracted enamel proteins at room temperature, while Parker *et al*. incubated the samples at 56 °C for 60 min. Thus, to avoid an increase of the Gln deamidation rate, the extraction of enamel proteins at room or even lower temperature with diluted acid may be the best approach^22^. Moreover, calculating the rate of Gln deamidation by integrated peak areas rather than measured ion intensities (as^11^) may yield better results in terms of estimation^22^. However, further work on amelogenin Gln deamidation is required to properly address the abovementioned issues.

## Discussion

We confidently identified the sex of all the individuals considered in this study, using enamel peptides. Individual 3_CM12-2, classified as a female by osteology, revealed the presence of AMELY within his enamel proteome, a robust marker of the male sex. Given that this individual was a young adult (~20 years) at the time of death, we suggest that the skeletal misclassification was due to his age and the related low degree of sexualization of the dimorphic districts. Such evidence clearly supports the need of alternative cost-effective methods for sex classification for doubtful skeletal materials. Compared to Stewart *et al*.^7^, we propose the use of at least two additional peptides/peaks together with *m/z* 440.2233 (SM(ox)IRPPY) to confidently identify AMELY as, for instance, *m/z* 432.2258 (SMIRPPY) and 396.7073 (M(ox)IRPPY) or other AMELY unique peptides (see Table S2). The sequence of the peptides has been further confirmed by MS^2^ spectra annotation.

The ‘Lovers of Modena’ have been recognized both as males by the presence of AMELY. The literature lacks of any comparable evidence (namely, hand-in-hand males) in terms of analogous geo-chronological contexts. Furthermore, to our knowledge, such a gesture was uncommon if not totally unrepresented in the art of Late Antiquity or, in general, before modern times. However, other archaeological sites, both geographically and temporally dispersed, yielded burials containing embraced or hand-in-hand individuals (see^23^ and references therein). This is the case, for instance, of the ‘Lovers of Valdaro’, a young female-male couple found embraced within a Neolithic tomb (San Giorgio, Mantua, Italy; ~6000 years BP). Of similar age (~5800 years BP) are the two embraced individuals discovered within the Greek site of Alepotrypa Cave. Similarly, two skeletons (male and female) were found embraced within a Turkish archaeological site (Diyarbakir, Turkey) dated to Neolithic times (~8000 years BP). The Bronze Age site of Staryi Tartas (Novosibirsk, Siberia) yielded several tombs containing female-male couples or, in some cases, even an entire family (two adults and two juveniles). The archaeologists suggest that these Bronze Age burials could be related to ritual sacrifices or to after-death beliefs^23^. In Romania, two skeletons dated to the Middle Ages (15th–16th century AD) were found buried hand-in-hand (Cluj-Napoca, Romania) within the cemetery of a Dominican convent^23^. In all cases, the burials seemed to contain female-male couples, even though a more robust sex determination by enamel proteins or DNA might be necessary to confidently classify the individuals.

We suggest that the ‘Lovers of Modena’ burial represents a voluntary expression of commitment between two individuals, rather than a recurring cult practice of the Late Antiquity; their position may reflect such relationship. The presence of several injured individuals within the Ciro Menotti necropolis let us suppose the destination of this place as war-cemetery^12^. In this sense, the two ‘Lovers’ could have been war comrades or friends, died together during a skirmish and, thus, buried within the same grave. Alternatively, the two individuals were relatives, possibly cousins or brothers given their similar ages, sharing the same grave due to their family bond. Although we cannot exclude that these two individuals were actually in love, it is unlikely that people who buried them decided to show such bond by positioning their bodies hand in hand. Particularly, Late Antique social attitudes and Christian religious restrictions lead to the rejection of any hypothesis of deliberate manifestation of homosexual relationship. In fact, since 390, male passivity was frowned upon by law^24^ and, during the reign of Justinian (527–565), sex between males was fully considered a crime^24^.

With this paper, we demonstrated that LC-MS/MS analysis of enamel peptides represents a robust method to estimate the sex of both ancient and modern human skeletal remains, ultimately providing an excellent contribution to paleoanthropology, bioarchaeology, and forensic anthropology. We also strengthened the method of Stewart *et al*.^7^, by proposing the use of three extracted ion chromatograms, corresponding to different AMELY unique peptides, to confidently identify protein AMELY. We applied this approach to a peculiar archaeological context, determining the sex of the ‘Lovers of Modena’, which turned out to be both males. These individuals are, thus, a unique representation of commitment between two men during the Italian Late Antiquity.

## Methods

### Sampling and pretreatment

The entire analytical protocol is summarized in Fig. S20. All reagents employed were of analytical grade, with the exception of hydrochloric acid being suprapur grade. Ultrapure water (18.2 MΩcm^−1^) was obtained from a Millipore water purification system (MilliQ Merck Millipore, Italy). From each skeletonized individual (n = 14), a tooth was extracted from the mandible or the maxilla using pliers. Two modern deciduous teeth were from personal collections and belonged to two of the authors of this paper. To improve the protein yield from the enamel tissue, we sampled small enamel chunks (ca. 10 mg) using a dentist drill (cleaned with 70% ethanol after each sampling), rather than extracting proteins by acid etch^7,20^. By employing this protocol, we were able to properly clean the sample surface before the actual extraction (see below) and to analyze the samples by LC-MS/MS. In comparison with the method proposed by Stewart *et al*.^7^, our protocol uses a traditional UPLC-MS/MS set up (advantage), but requires, at this stage, more material for the analysis (disadvantage). Enamel chunks were rinsed with MilliQ water in an ultrasonic bath, leached for 5 minutes with 200 μL of 5% HCl and then re-washed with MilliQ.

### Enamel protein extraction and purification

To extract enamel proteins, each specimen was soaked in 200 μL of 5% HCl for 1 h in an Eppendorf tube at room temperature. After 1 h, none of the samples (ca. 10 mg of enamel) was completely digested. The supernatant was collected in a new Eppendorf tube and peptides were extracted by HyperSep SpinTips (Thermo Scientific) with C_18_ functionalized silica, according to the manufacturer’s protocol. Briefly, tips were conditioned three times with 50 μL of 100% acetonitrile and three times with 50 μL of 0.1% formic acid. Samples (200 μL of HCl) were loaded on the SpinTips plug in two steps of 100 μL each. Tips were then washed three times using 50 μL of formic acid 0.1%. Resin-bounded proteins were finally eluted using 10 μL of 60% acetonitrile in 0.1% formic acid, repeating the elution step twice. Samples were dried down at room temperature under a laminar flow hood (class 100). All the previously described protocols were performed at the Department of Chemical and Geological Sciences (Cleanroom facility, class 1000) and the Department of Life Sciences (Bioinorganic Chemistry and Bioelectrochemistry Lab) of the University of Modena and Reggio Emilia.

### Tandem mass spectrometry

For UHPLC–HRMS analysis, dry extracted peptides were resuspended in 50 µL of a mixture of water:acetonitrile:formic acid 95:3:2, sonicated for 10 minutes at room temperature and centrifuged at 12100 r.c.f. for 10 minutes. A Thermo Scientific Dionex Ultimate 3000 UHPLC coupled to a Thermo high-resolution Q Exactive mass spectrometer (Thermo Scientific, Bremen, Germany) was used for the analyses. The column (Zorbax SB-C18 RRHT, 2.1 × 50 mm, 1.8 μ particle size, Agilent Technologies), thermostatted at 25 °C, was equilibrated with 0.3 mL/min of water 0.1% formic acid (A) with 2% acetonitrile (B); after sample injection (15 µl), B% was kept constant at 2% for 2′, then linearly increased from 2 to 28% in 64 minutes; B% was then brought to 95% in 4 minutes and kept at 95% B for 5 minutes, before the reconditioning step. Each sample required a total run time of 90 minutes. Centroided MS and MS^2^ spectra were recorded from 200 to 2000 m/z in Full MS/dd-MS² (TOP2) mode, at a resolution of 35000 and 17500, respectively. The two most intense multi-charged ions (TOP2) were selected for MS^2^ nitrogen-promoted collision-induced dissociation (NCE = 28). Precursor dynamic exclusion (6 seconds) and apex triggering (1 to 5 s) were set; peptide-like isotope pattern ions were preferred. The mass spectrometer was calibrated before the start of the analyses; an initial segment (0.1–0.7 minutes) with a lock mass (391.28429, corresponding to the [MH]^+^ of diisooctyl phtalate, an ubiquitous contaminant) was included in the MS method. More details are reported within the Supplementary Information.

### Database search

For protein identification, raw data, converted into mascot generic format using MsConvert (v. 3.0.10730, ProteoWizard tools^25^), were searched against Swiss-Prot (accessed Oct 2018; 20350 sequences for *Homo Sapiens*) for peptide sequences and cRAP (116 sequences) for contaminants with Mascot Server (Version 2.4, Matrix Science, London, UK). No proteolytic enzyme was selected, deamidated asparagines/glutamine (NQ) and oxidated methionine (M) were set as variable modifications in the search parameters. One missed cleavage was allowed. Mass tolerances were set at 10 ppm for the precursor ions (peak detection mismatch #^13^C = 1) and 0.05 Da for the product ions. An automatic decoy database search was used to estimate the false discovery rate; probability threshold was trimmed to get a FDR < 1%.

### Glutamine deamidation rate

Deamidation rate of glutamine (deQ) was calculated from peptides of the AMELX protein containing sequence YQSIRPPYP, *e.g*.: VLTPLKWYQSIRPPYP (non-deamidated [M + 3 H]^3+^ 653.3681 *m/z*; deamidated [M + 3 H]^3+^ 653.6961 *m/z*); YEVLTPLKWYQSIRPPYP (non-deamidated [M + 3 H]^3+^ 750.7367 *m/z*; deamidated [M + 3 H]^3+^ 751.0648 *m/z*); YEVLTPLKWYQSIRPPYPS (non-deamidated [M + 3 H]^3+^ 834.1019 *m/z*; deamidated [M + 3 H]^3+^ 834.4299 *m/z*); YEVLTPLKWYQSIRPPYPSYG (non-deamidated [M + 3 H]^3+^ 853.1090 *m/z*; deamidated [M + 3 H]^3+^ 853.4370 *m/z*). The proportion of Gln deamidation (%deQ) was calculated as follows (see^11^):

$$ \% {\rm{deQ}}=\frac{I{P}_{deQ}}{(I{P}_{deQ}+I{P}_{ndeQ})}$$

where IP_ndeQ_ is the chromatogram integrated area of the peptide peak containing non-deamidated Gln and IP_deQ_ is the integrated area of the peptide peak containing deamidated Gln, namely Glu. Integrated peak areas were calculated using Qual Browser (Thermo Scientific Xcalibur). At last, an average value (±SD) was calculated for each sample, combining the different deamidation rates of Gln for the different peptides. A %deQ of 0 represents a not deamidated specimen, while a %deQ of 1 represents a completely deamidated specimen.

### Blinding

Mass spectrometry experiments were blinded: sample selection was performed by one of the authors of the paper (GG), assigning random numbers to the specimens, and then employed as reference numbers for the sample preparation and MS analysis. The subsequent LC-MS/MS analyses and chromatogram interpretations were performed without any prior knowledge about the skeletal sex of the individual. Only at the end of the analysis, the results about the sex of the individuals were cross-checked.

## Supplementary information

Supplementary Information

## Data Availability

All data generated or analyzed during this study are included in this paper (and its Supplementary Information Files). The mass spectrometry proteomics raw data have been deposited to the ProteomeXchange Consortium via the PRIDE^26^ partner repository with the dataset identifier PXD012587.
