# Supplementary material for: Enamel peptides reveal the sex of the Late Antique ‘Lovers of Modena’
Source: Sci Rep. 2019 Sep 11;9:13130. doi: 10.1038/s41598-019-49562-7 (PMC6739468; doi:10.1038/s41598-019-49562-7)
Supplement: Supplementary file 1 — Supplementary Information [file 41598_2019_49562_MOESM1_ESM.docx]

**Supplementary Information for:**

**Enamel peptides reveal the sex of the Late Antique ‘Lovers of Modena’**

Federico Lugli^a,b,^*, Giulia Di Rocco^c,^*, Antonino Vazzana^a^, Filippo Genovese^d^, Diego Pinetti^d^, Elisabetta Cilli^a^, Maria Cristina Carile^a^, Sara Silvestrini^a^, Gaia Gabanini^a^, Simona Arrighi^a^, Laura Buti^a^, Eugenio Bortolini^a^, Anna Cipriani^b,e^, Carla Figus^a^, Giulia Marciani^a^, Gregorio Oxilia^a^, Matteo Romandini^a^, Rita Sorrentino^a,f^, Marco Sola^c^, Stefano Benazzi^a,g^

^a^Department of Cultural Heritage, University of Bologna, Via degli Ariani 1 ‒ 48121 Ravenna, Italy

^b^Department of Chemical and Geological Sciences, University of Modena and Reggio Emilia, Via Campi 103 – 41125 Modena, Italy

^c^Department of Life Sciences, University of Modena and Reggio Emilia, Via Campi 103 – 41125 Modena, Italy

^d^Centro Interdipartimentale Grandi Strumenti, University of Modena and Reggio Emilia, Via Campi 213/A – 41125 Modena, Italy

^e^Lamont-Doherty Earth Observatory, Columbia University, Palisades, New York, USA

^f^Department of Biological, Geological and Environmental Sciences, University of Bologna, 40126 - Bologna, Italy

^g^Department of Human Evolution, Max Planck Institute for Evolutionary Anthropology, Leipzig, Germany

*corresponding authors: [federico.lugli6@unibo.it](mailto:federico.lugli6@unibo.it); [giulia.dirocco@unimore.it](mailto:giulia.dirocco@unimore.it)


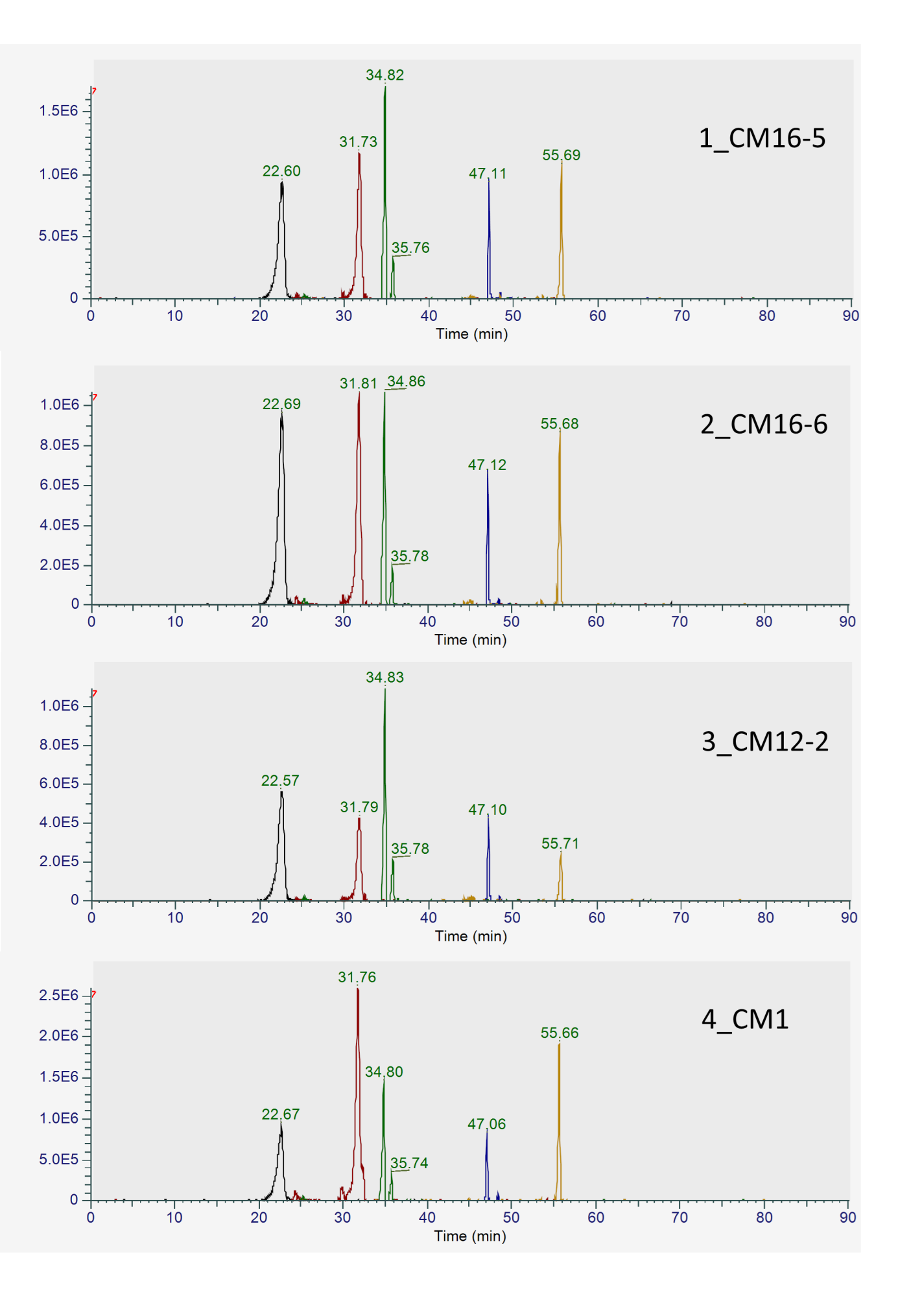

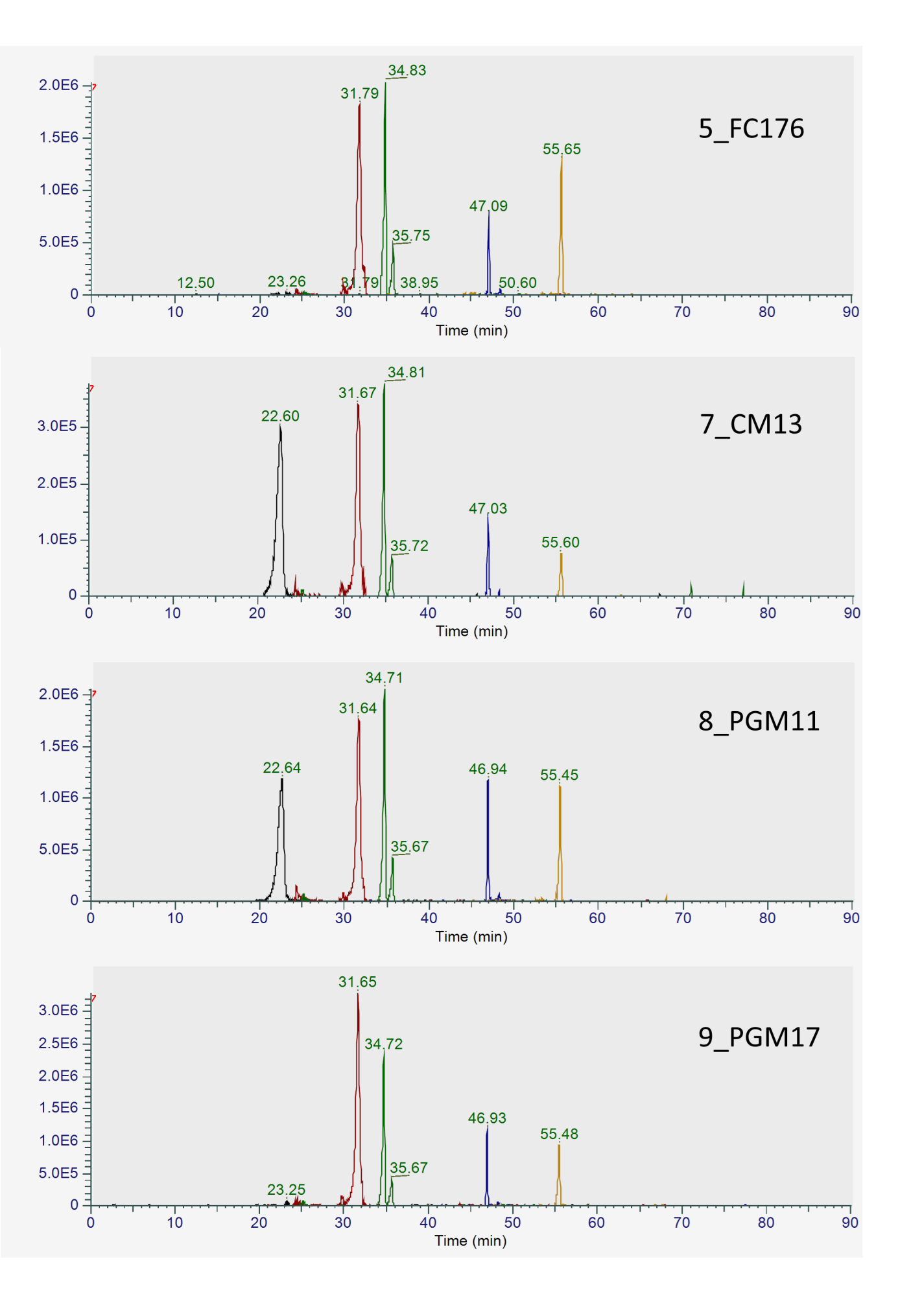

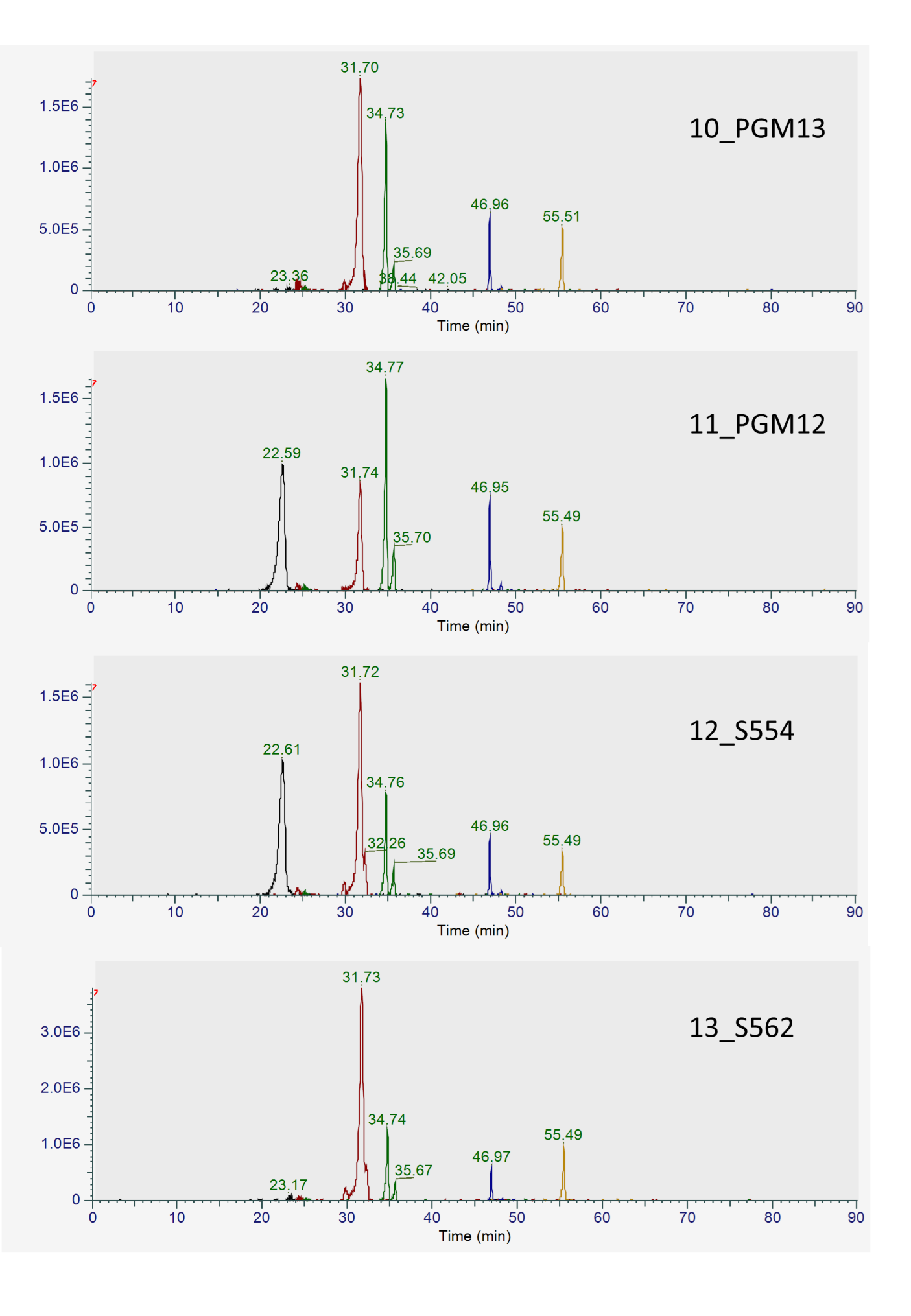

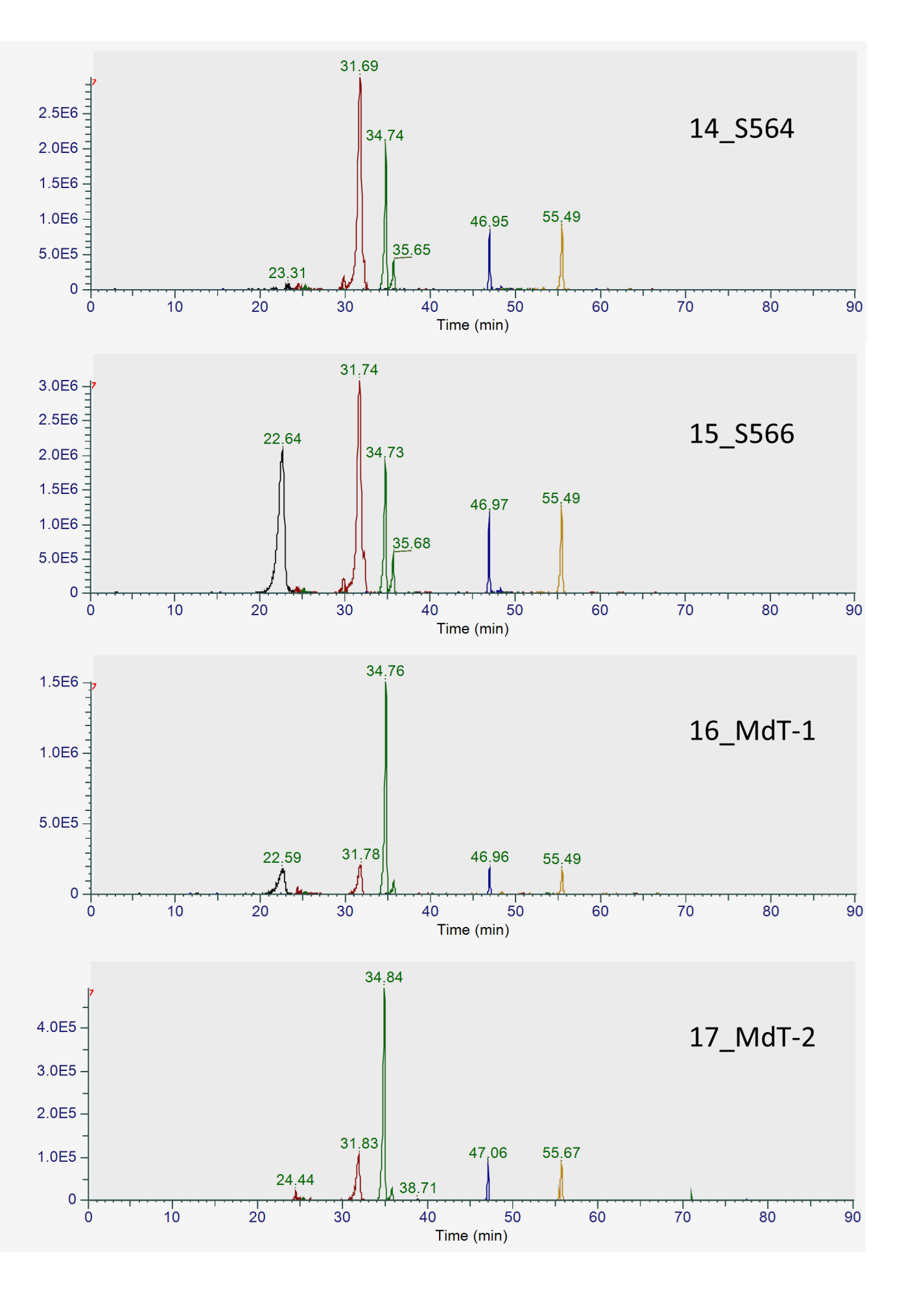


**Figures S1-S16.** Extracted ion chromatograms representing selected peptides of all the individuals analyzed in this study. Chromatograms extraction was performed using Xcalibur software (Thermo Scientific) with a mass tolerance of 5 ppm; y-axis is ion intensity. The black peak is peptide SM(ox)IRPPY (AMELY; [M+2H]^+2^  440.2233 *m/z*); the red peak is peptide SIRPPYPSY (AMELX; [M+2H]^+2^ 540.2796 *m/z*); the green peak is SYEVLTPLK (AMELX,Y; [M+2H]^+2^ 525.2975 *m/z*); the blue peak is PYFGYFGYH (ENAM; [M+2H]^+2^ 575.7533 *m/z*) and the orange peak is YEVLTPLKWY (AMELX,Y; [M+2H]^+2^ 656.3528 *m/z*). The presence of peptide SM(ox)IRPPY (AMELY; [M+2H]^+2^  440.2233 *m/z*; black peak) identifies male individuals. For the ID of each individual refer to Table 1 of the manuscript.

**Mass spectrometry**

For UPLC–HRMS analysis, dry extracted peptides were resuspended in 50 µL of a mixture of water:acetonitrile:formic acid 95:3:2, sonicated for 10 minutes at room temperature and centrifuged at 12'100 r.c.f. for 10 minutes. 15 µL of this solution were injected in the instrument. A Thermo Scientific Dionex Ultimate 3000 UHPLC system equipped with a Binary HPG 3400RS Pump and a Thermo Scientific Dionex Ultimate 3000 Series TCC-3000RS column compartments with a Thermo Fisher Scientific Ultimate 3000 Series WPS-3000RS autosampler controlled by Chromeleon 7.2 Software (Thermo Fisher Scientific, Waltham, MA and Dionex Softron GmbH, part of Thermo Fisher Scientific, Germany) hyphenated with a Thermo high resolution Q Exactive mass spectrometer (Thermo Scientific, Bremen, Germany) were used for analysis. Nitrogen (purity > 99.999%), obtained from a Zefiro zero 60 LC-MS nitrogen generator (CINEL, Vigonza, Italy), was employed as both the collision and source gas. The chromatographic system was coupled to the MS with a Heated Electrospray Ionization Source II (HESI II). The column (Zorbax SB-C18 RRHT, 2.1x50mm, 1.8 μ particle size, Agilent Technologies), thermostatted at 25°C, was equilibrated with 0.3 ml/min of water 0.1% formic acid (A) with 2% acetonitrile (B); after sample injection (15 µl), B% was kept constant at 2% for 2’, then linearly increased from 2→28% in 64 minutes; B% was then brought to 95% in 4 minutes and kept at 95% B for five minutes, before the reconditioning step. The total runtime was 90 minutes.

ESI source was operated in positive mode; probe was heated at 290°C, capillary temperature was set at 270 °C; the following nitrogen flows (arbitrary units) were used to assist the ionization: Sheath Gas 40, Aux Gas 30, Sweep Gas 3; capillary voltage was set to 3.8 kV, S-Lens RF level was set at 45 (arbitrary units).

Centroided MS and MS^2^ spectra were recorded from 200 to 2000 m/z in Full MS/dd-MS² (TOP2) mode, at a resolution of 35000 and 17500, respectively. The two most intense multi-charged ions (TOP2) were selected for MS^2^ nitrogen-promoted collision-induced dissociation (NCE=28). Precursor dynamic exclusion (6 seconds) and apex triggering (1 to 5s) were set; peptide-like isotope pattern ions were preferred. An inclusion list comprising 6 entries with the m/z and possible charge states of the peptides of interest was included in the method ([M+2H]^+2^ 523.7748; 440.2233; 540.2796; 525.2975; 575.7533; 656.3528 *m/z*). The mass spectrometer was calibrated before the start of the analyses; an initial segment (0.1-0.7 minutes) with a lock mass (391.28429) was included in the MS method. FreeStyle (v. 1.3, Thermo Fisher Scientific, San José, CA, USA) was used for data processing.

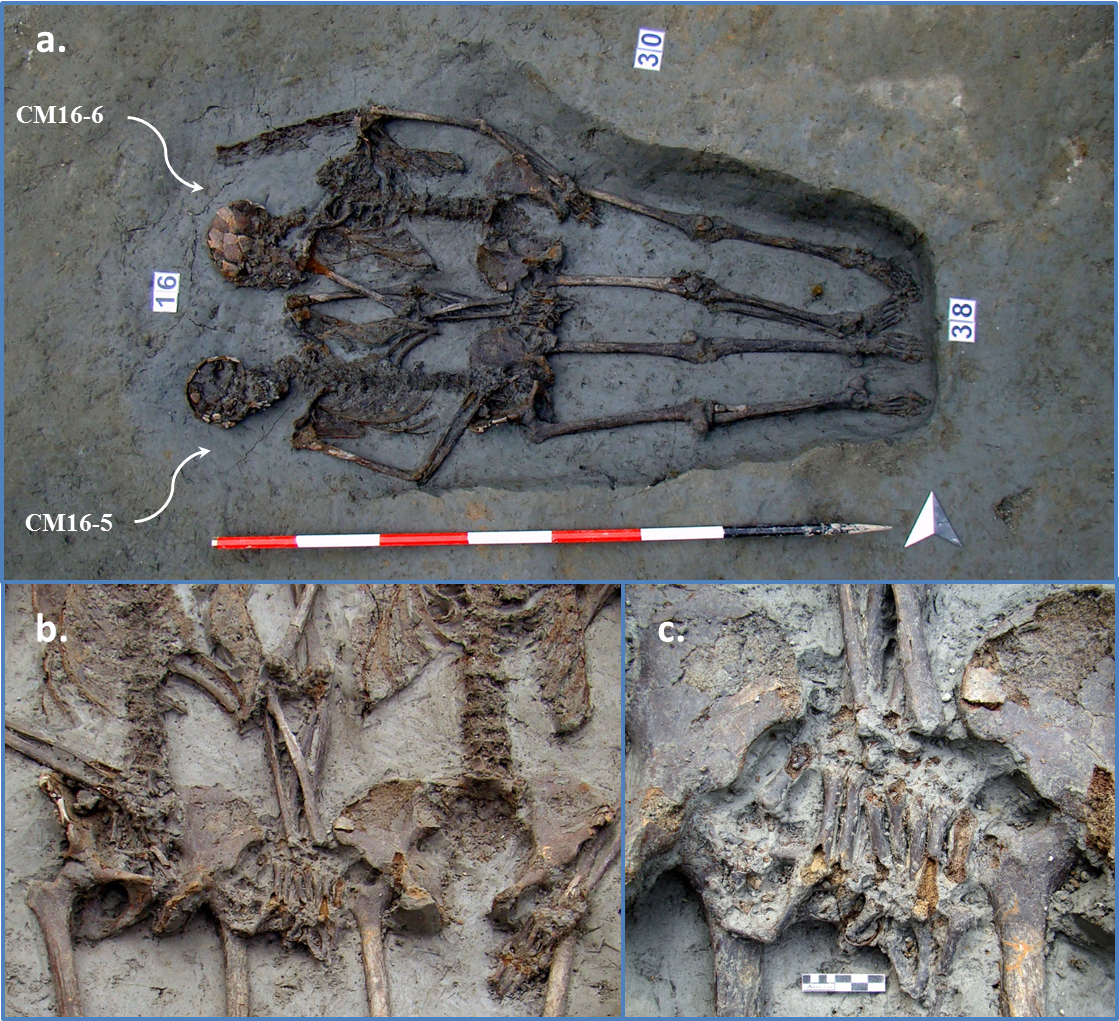


Figure S17. (a) Picture of the ‘Lovers of Modena’ (Ciro Menotti, Modena, 4^th^ – 6^th^ century AD); (b and c) details of the hands of the two individuals. Taphonomic observations suggest that the peculiar deposition of the ‘Lovers’ was intentional.

**Taphonomy**

The retention of the anatomical connection of diarthrosis joints indicates that the decomposition of the two individuals occurred in filled space. Thus, we hypothesize that the relative positions of the skeletons was preserved since the moment of their burial. The observation of the skeleton deposition also suggests that the inhumation of the two individuals was coeval. Individual CM16-5 was deposited first and CM16-6 afterwards, with the right arm of the latter that directly overlies the left arm of the former. Observing the bone arrangement of CM16-5, the left forearm seems intentionally rotated in supination, with the palm of the hand disposed upwards. In contrast, the right hand and forearm of individual CM16-6 were pronated: the palm of the hand was thus rotated downwards, in contact with the palm of CM16-5 left hand.

Duday, H., Courtaud, P., Crubezy, E., Sellier, P., & Tillier, A. M. (1990). L'Anthropologie «de terrain»: reconnaissance et interprétation des gestes funéraires. Bulletins et Mémoires de la Société d'Anthropologie de Paris, 2(3), 29-49.


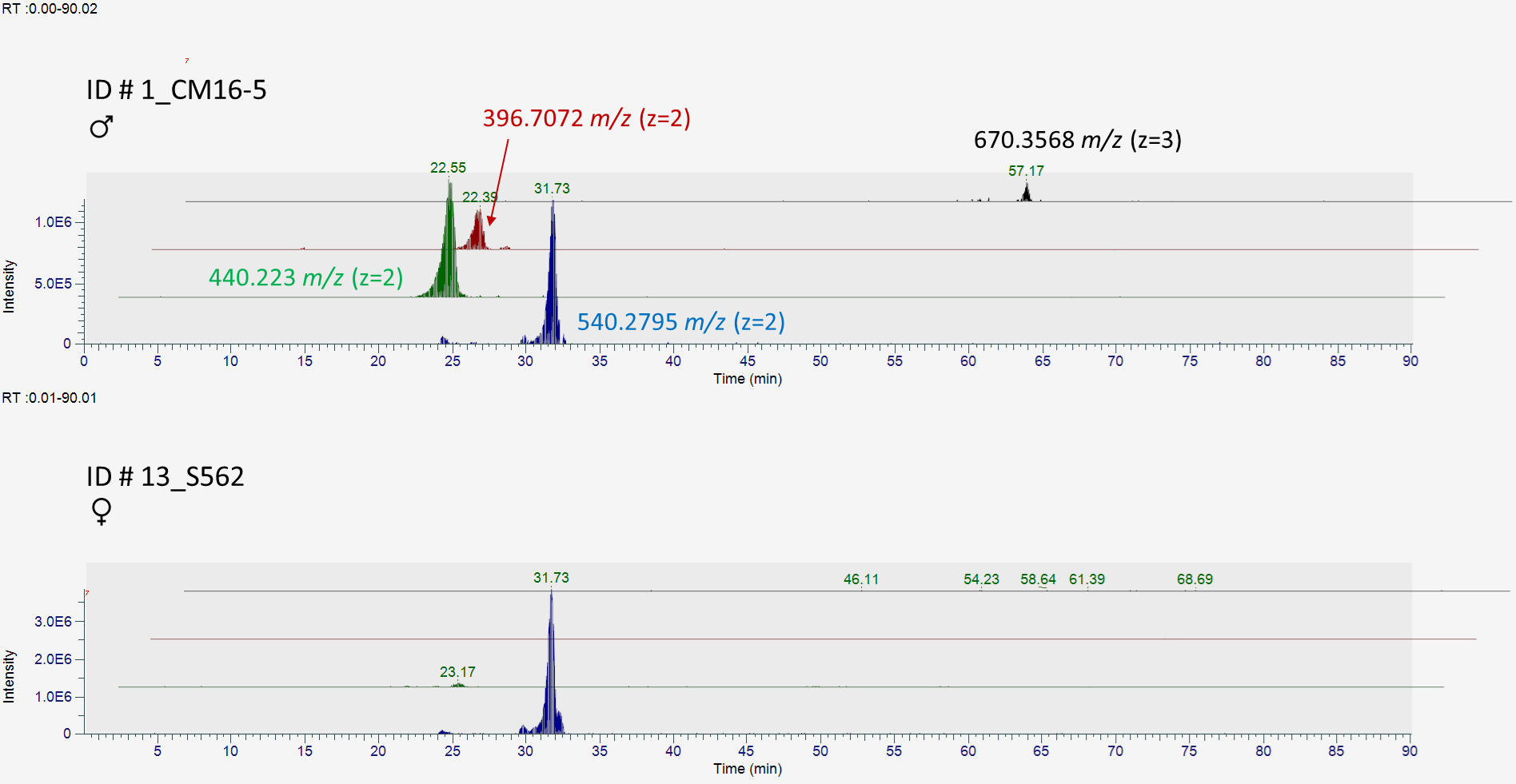


**Figure S18.** Extracted ion chromatograms of individuals 1_CM16-5 (male) and 13_S562 (female). Male-specific peptides (Table S2) were searched within the chromatograms. Peptide 540.2795 m/z (z = 2) is related to AMELX and reported as comparison.


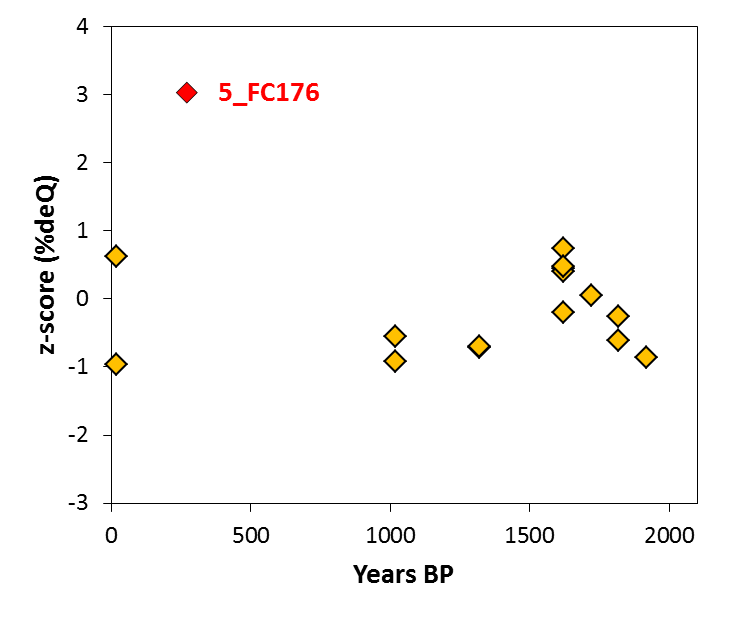


Figure S19. Z-scores calculated from the residuals of the %deQ log model (see main text) and plotted vs. the age of the sample. Specimen 5_FC176 falls at 3SD from the mean value.


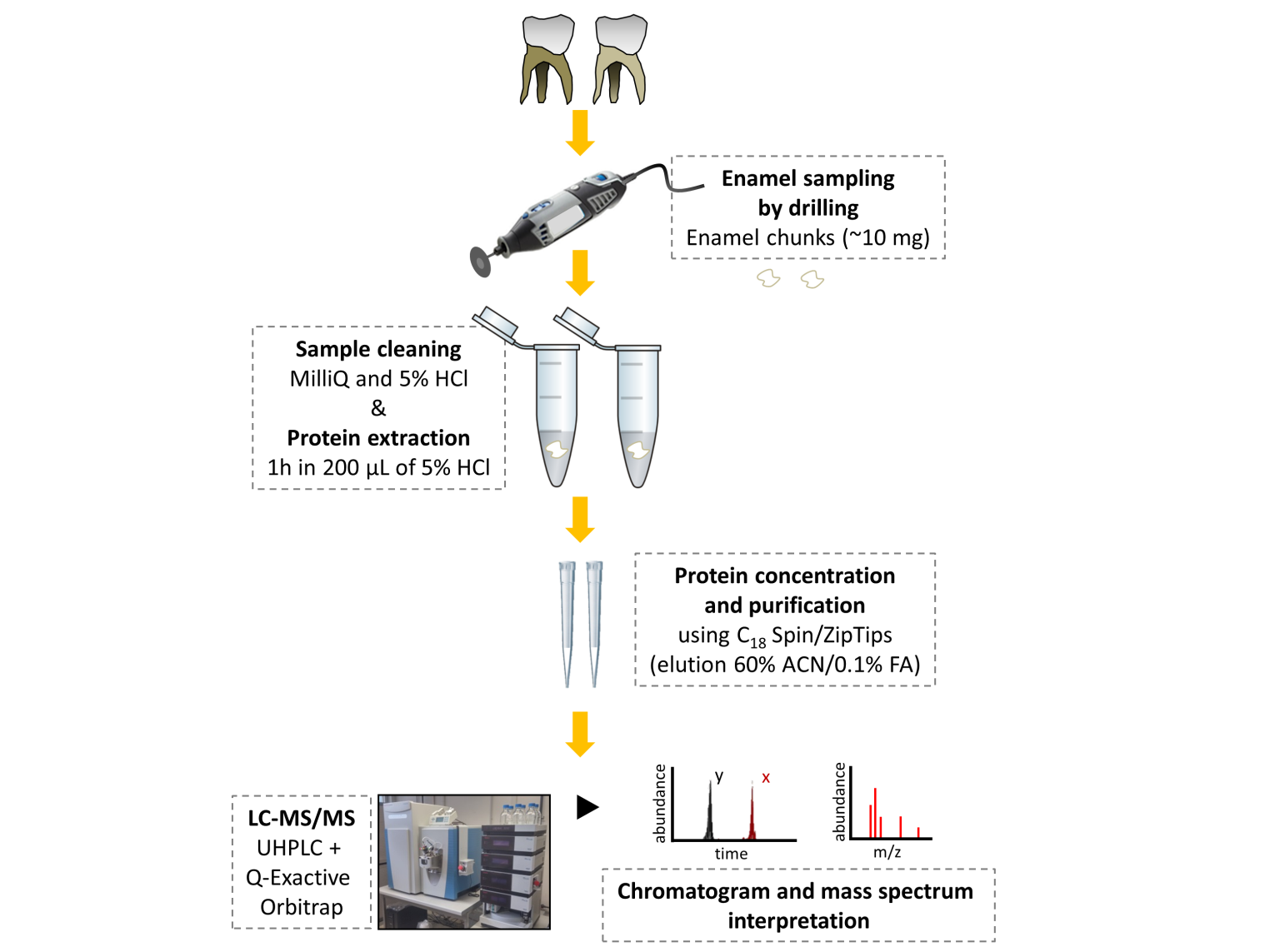


Figure S20. The analytical workflow employed in this work.


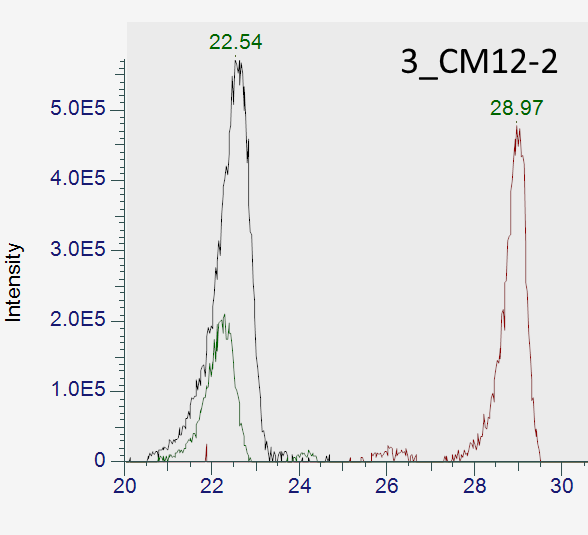


Figure S21. Ion chromatogram representing peptides SM(ox)IRPPY, M(ox)IRPPY and SMIRPPY of individual 3_CM12-2 (see Table 1 and Figure 3 of the main manuscript).


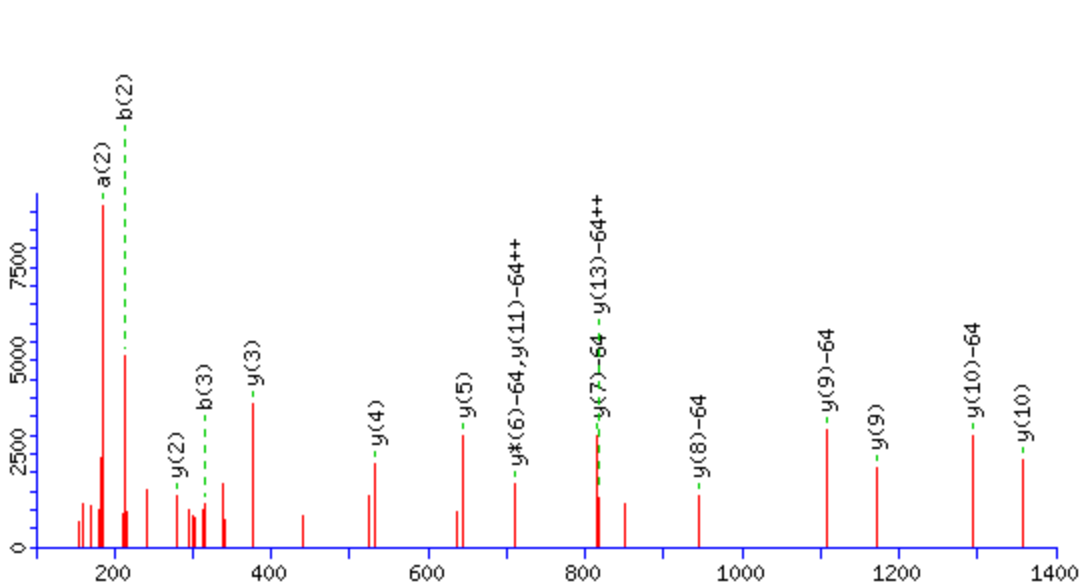


**VLTPLKWYQSM(ox)IRPPY**

Mascot score: 51

ID: 1_CM16-5


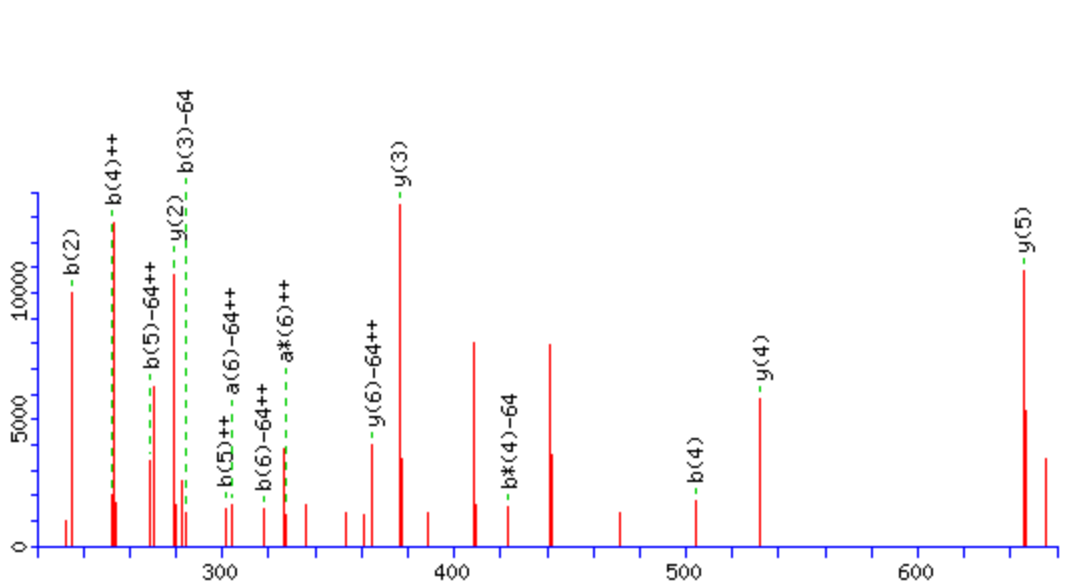


**SM(ox)IRPPY**

Mascot score: 26

ID: 1_CM16-5


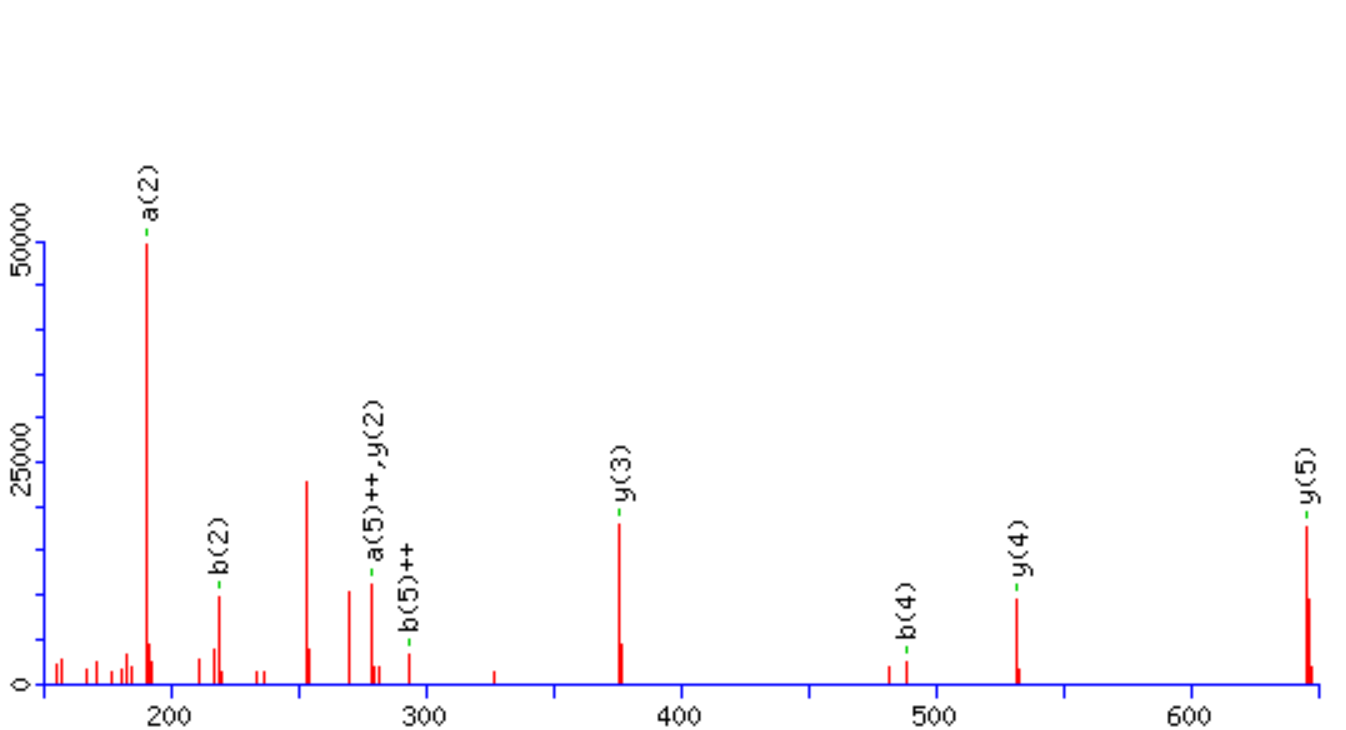


**SMIRPPY**

Mascot score: 32

ID: 1_CM16-5


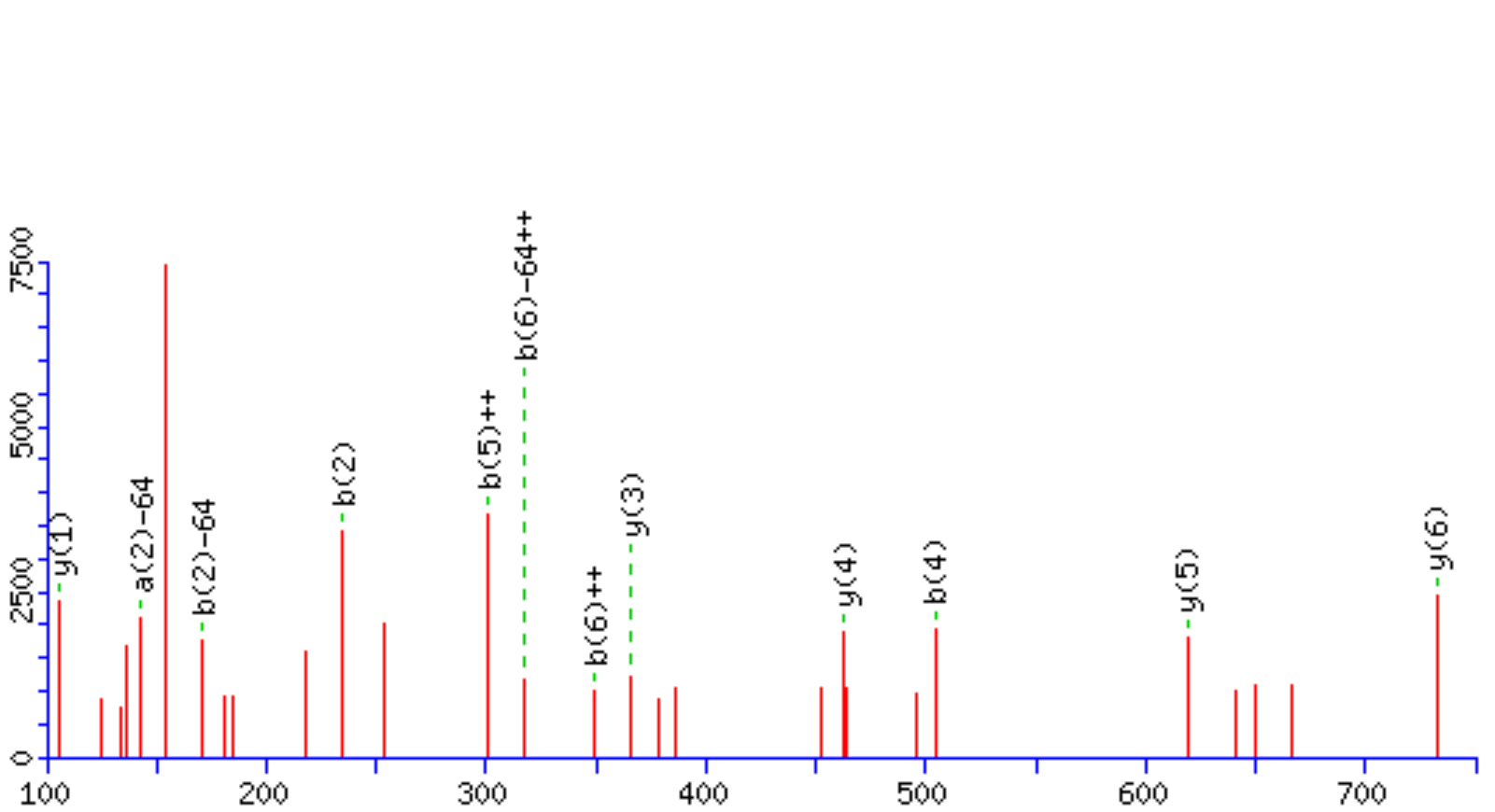


**SM(ox)IRPPYS**

Mascot score: 29

ID: 1_CM16-5


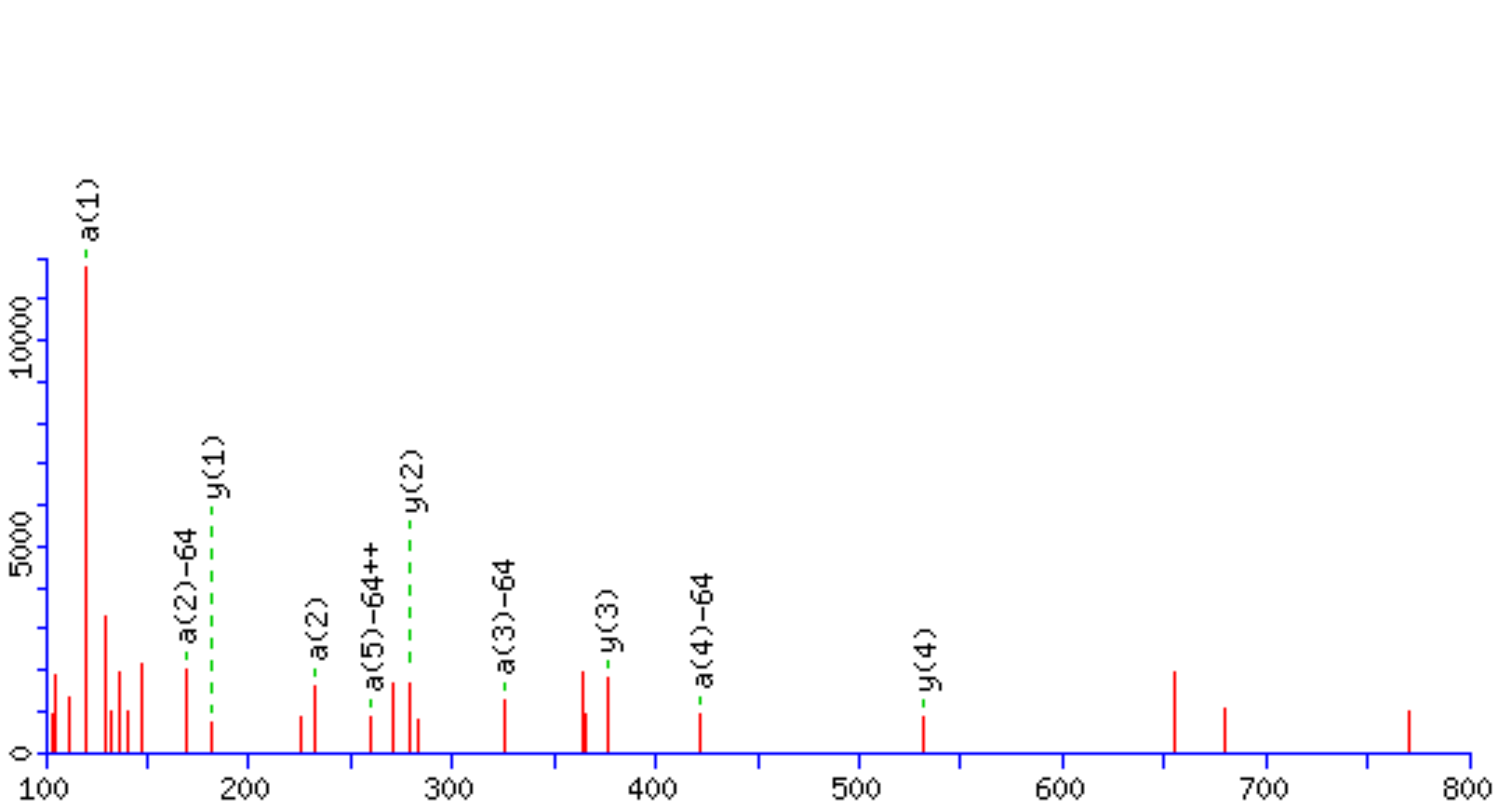


**M(ox)IRPPY**

Mascot score: 21

ID: 1_CM16-5


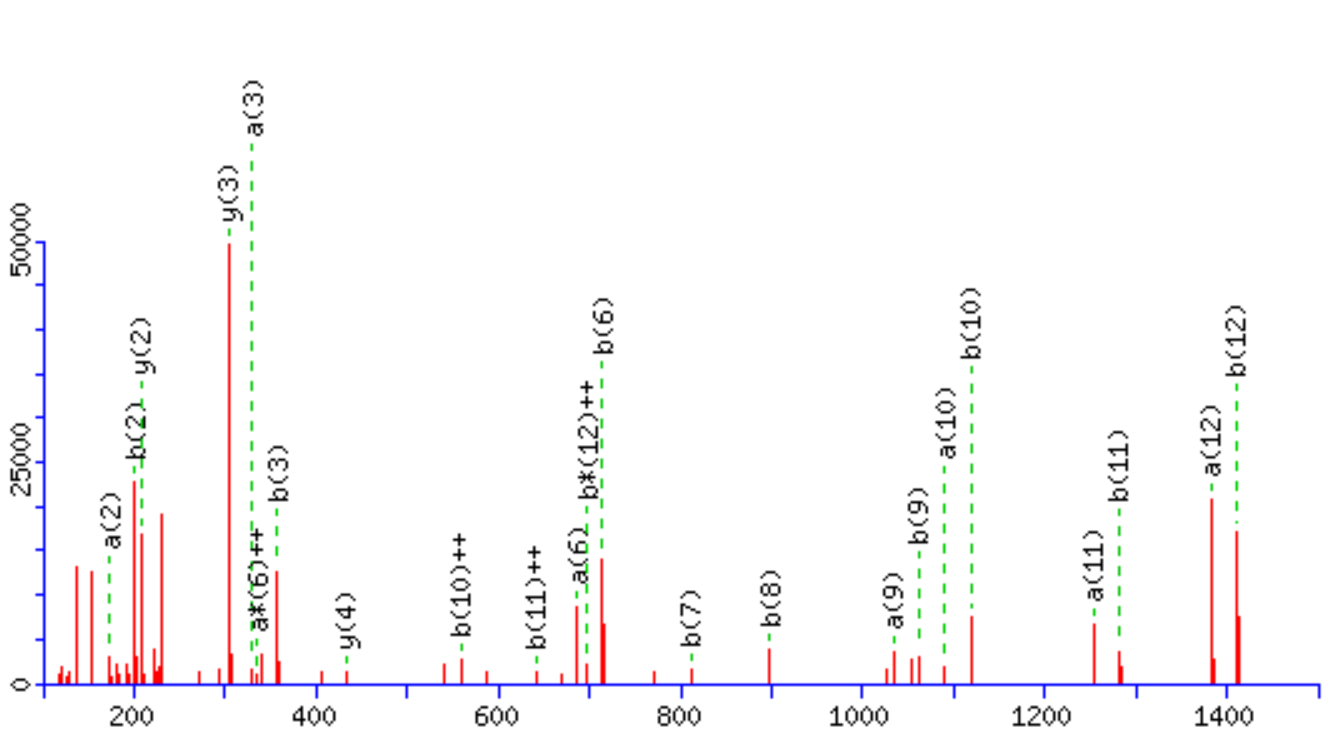


**SIRPPYPSYGYEPMG**

Mascot score: 50

ID: 1_CM16-5


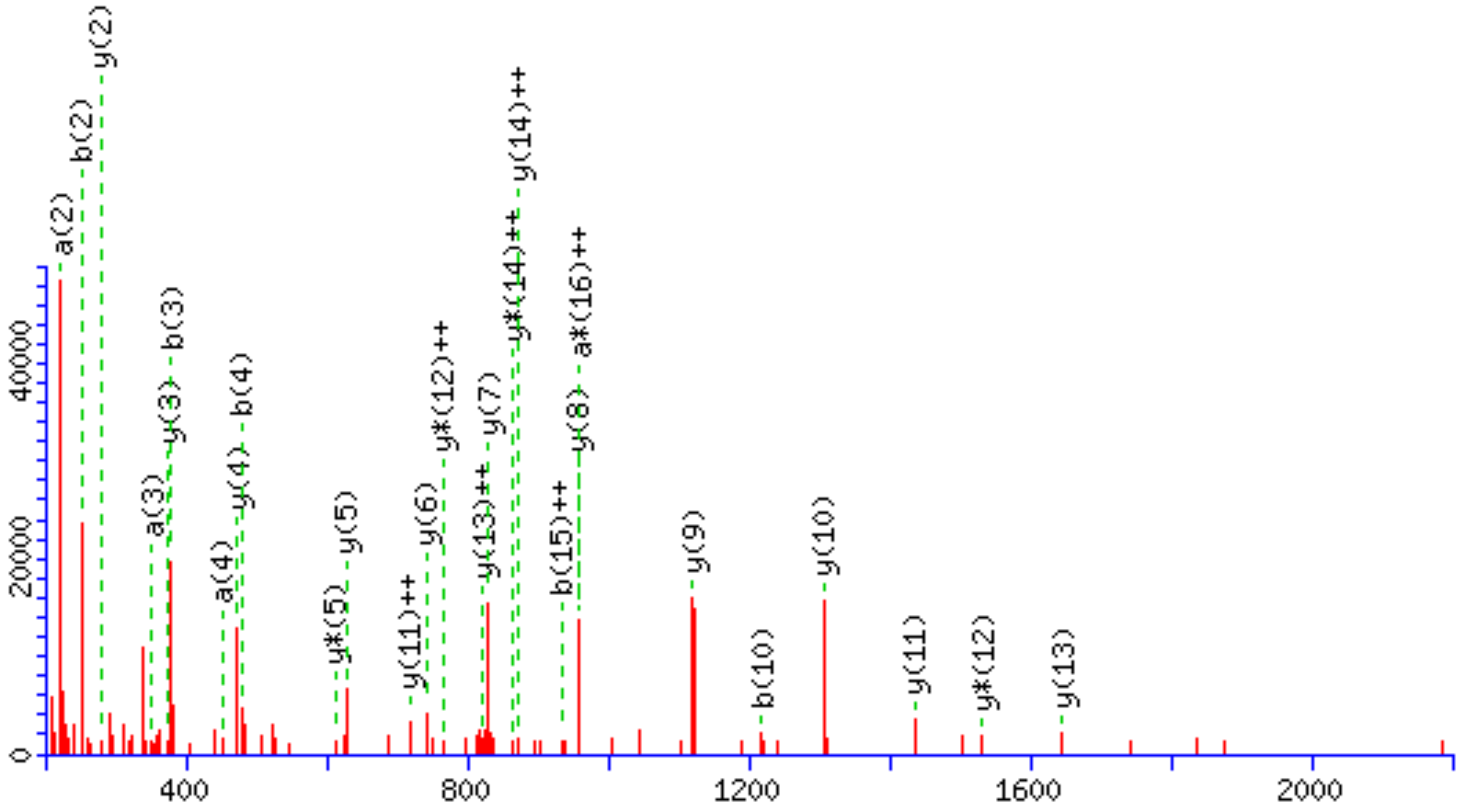


**SYEVLTPLKWYQSIRPPYP**

Mascot score: 49

ID: 1_CM16-5


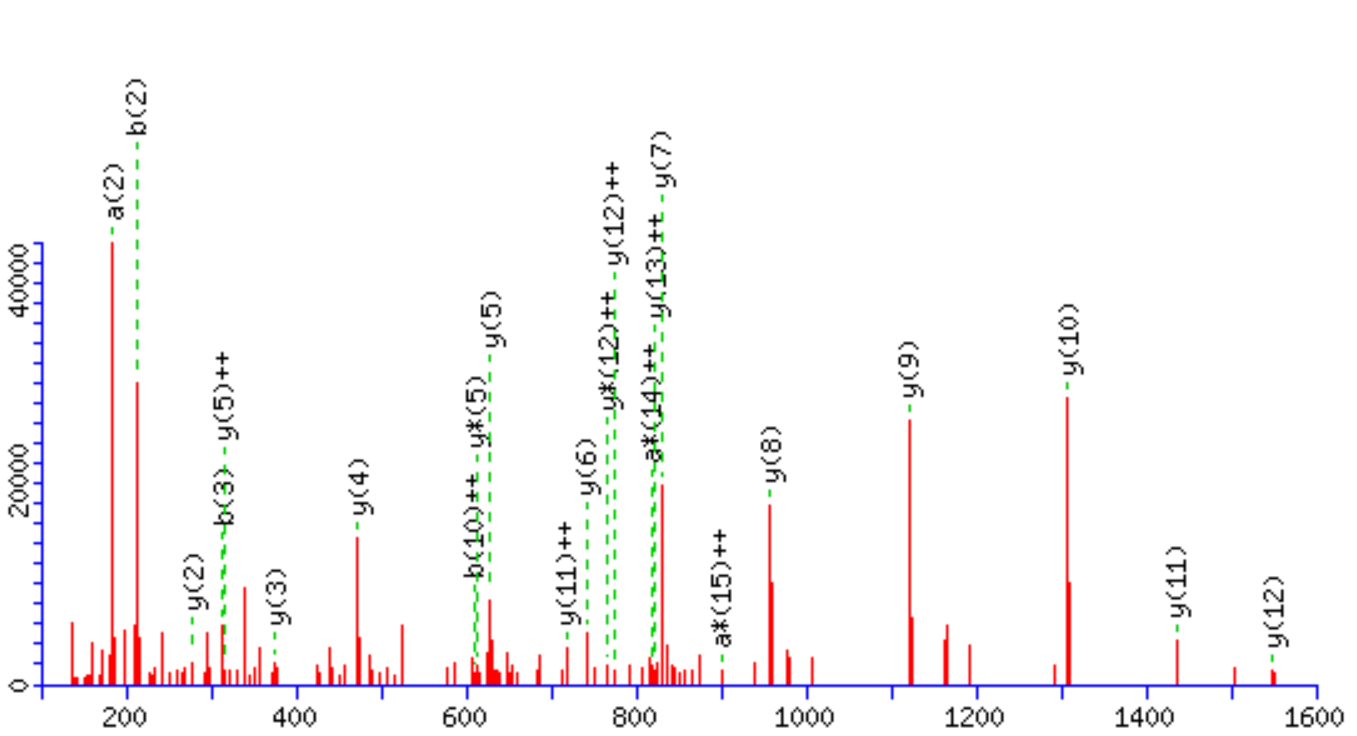


**VLTPLKWYQSIRPPYP**

Mascot score: 56

ID: 1_CM16-5


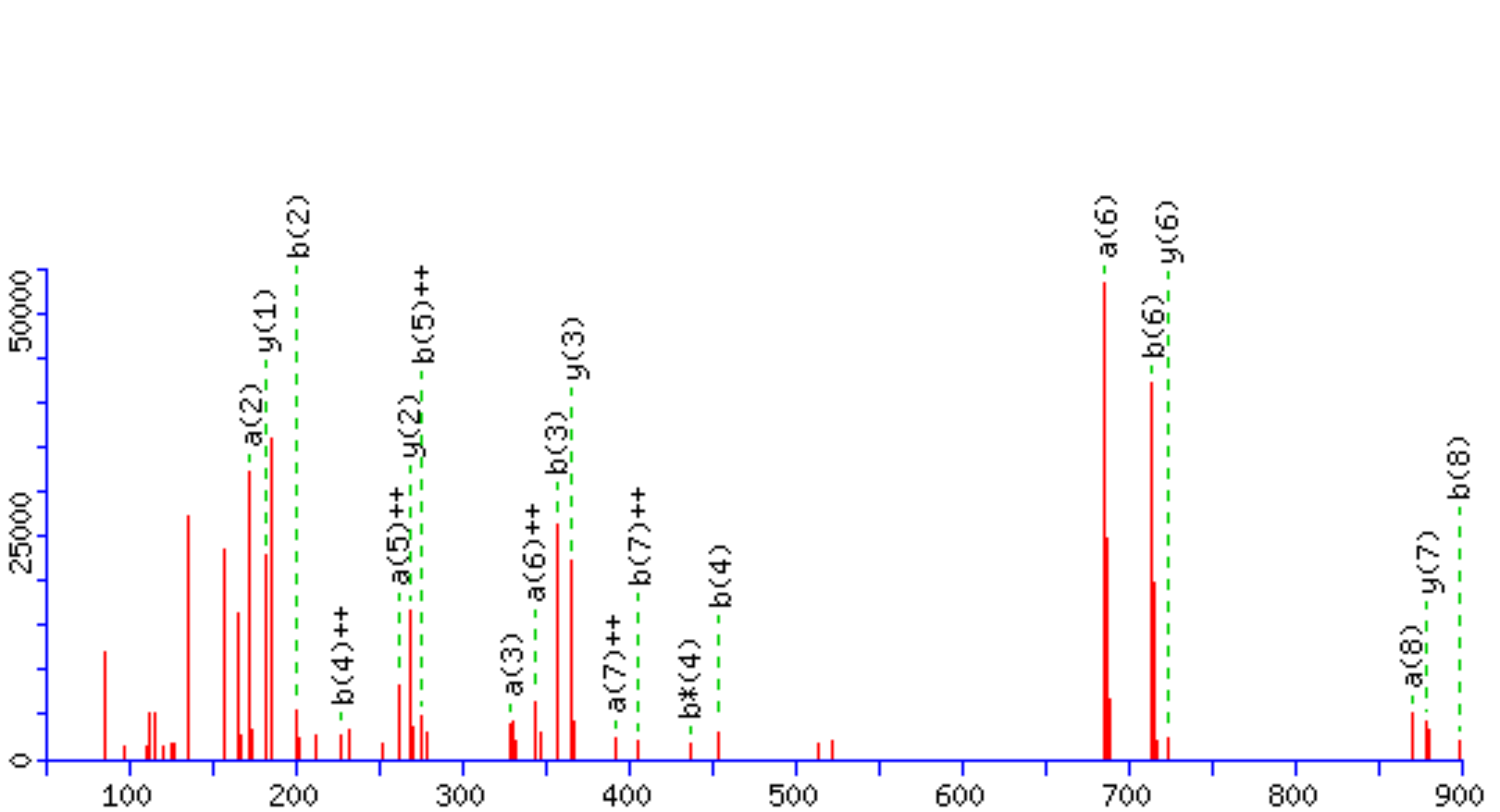


**SIRPPYPSY**

Mascot score: 30

ID: 1_CM16-5

**Figures S22-S30.** MS^2^ fully annotated spectra of relevant peptides (see Table S2) from Mascot searches. Y-axis indicates ion intensities; while x-axis reports m/z.


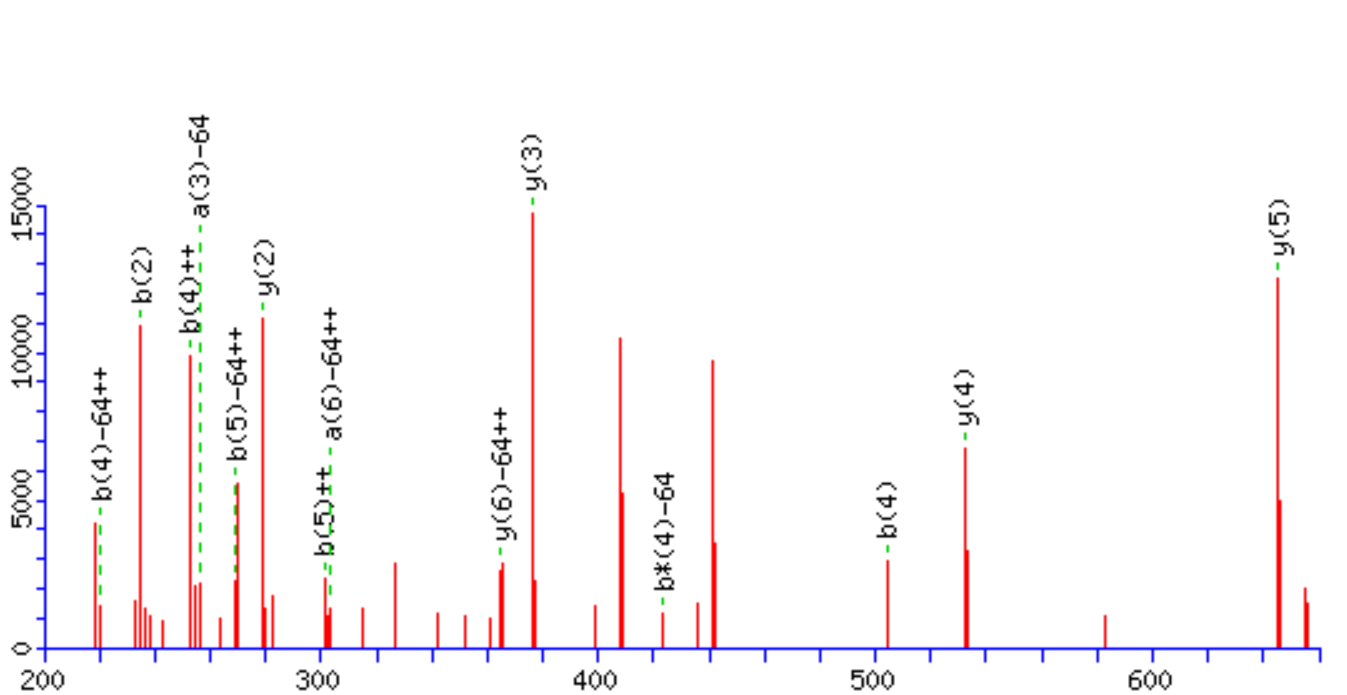


**SM(ox)IRPPY**

Mascot score: 31

ID: 2_CM16-6


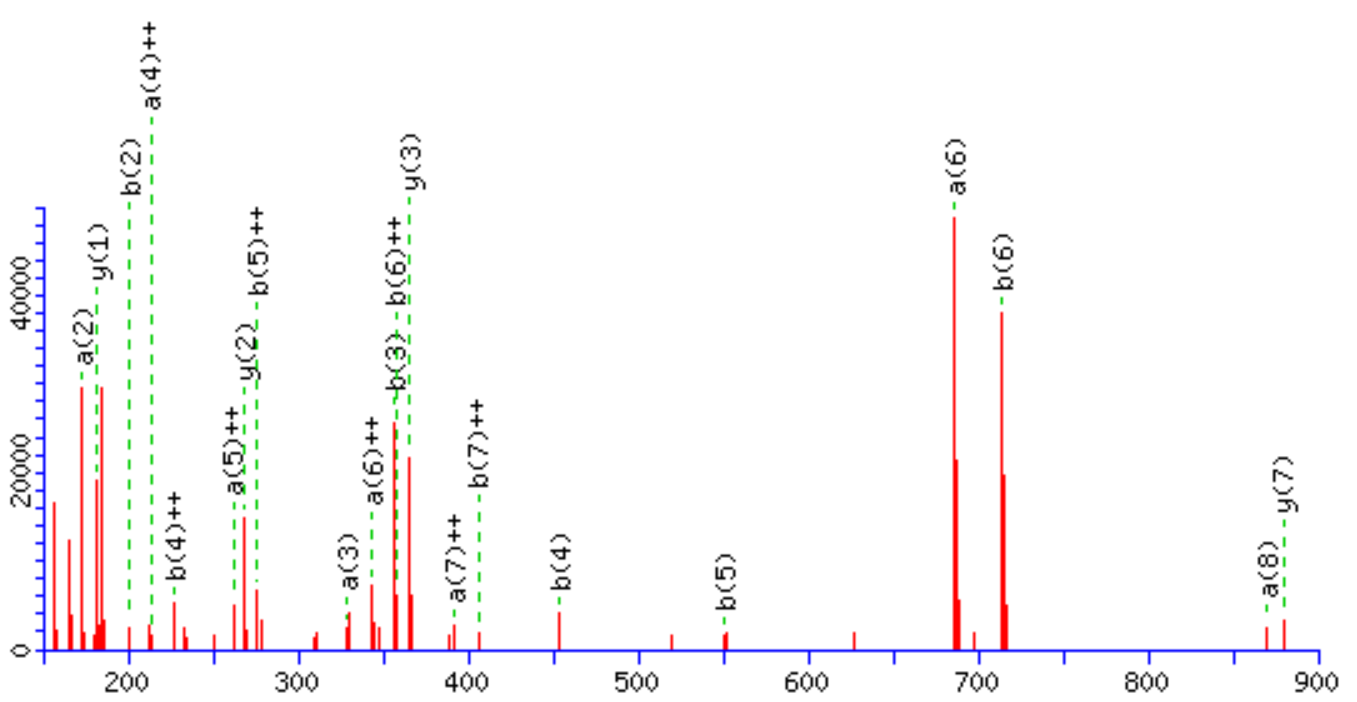


**SIRPPYPSY**

Mascot score: 32

ID: 2_CM16-6

**Figures S31-S32.** MS^2^ fully annotated spectra of individual 2_CM16-6. Y-axis indicates ion intensities; while x-axis reports m/z.
